# A strained alkyne-containing bipyridine reagent; synthesis, reactivity and fluorescence properties†

**DOI:** 10.1039/c9ra06866j

**Published:** 2019-11-06

**Authors:** Sam Forshaw, Richard C. Knighton, Jami Reber, Jeremy S. Parker, Nikola P. Chmel, Martin Wills

**Affiliations:** Department of Chemistry, The University of Warwick Coventry CV4 7AL UK m.wills@warwick.ac.uk; Early Chemical Development, Pharmaceutical Sciences, IMED Biotech Unit, AstraZeneca Macclesfield SK10 2NA UK

## Abstract

We report the synthesis of a bipyridyl reagent containing a strained alkyne, which significantly restricts its flexibility. Upon strain-promoted alkyne-azide cycloaddition (SPAAC) with an azide, which does not require a Cu catalyst, the structure becomes significantly more flexible and an increase in fluorescence is observed. Upon addition of Zn(ii), the fluorescence is enhanced further. The reagent has the potential to act as a fluorescent labelling agent with azide-containing substrates, including biological molecules.

## Abstract 2

A bipyridyl reagent containing a strained alkyne 7, reacts with benzyl azide to give a significantly more flexible product 10 and an increase in fluorescence is observed. Upon addition of Zn(ii), the fluorescence is enhanced further.

## Graphical abstract

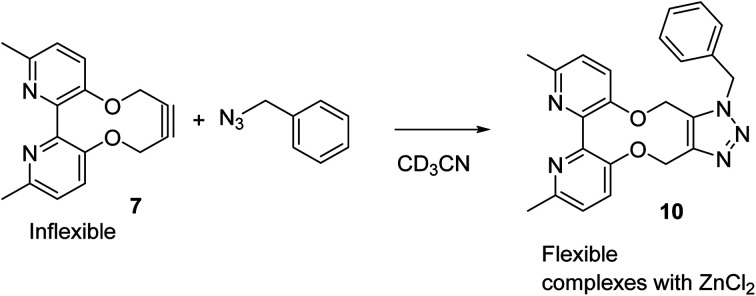

## Introduction

**Scheme 1:**
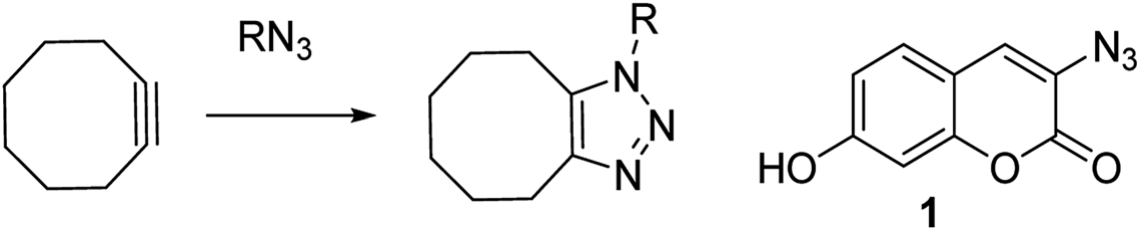
Uncatalysed reaction of azide with strained cyclooctyne, and a coumarin reagent which fluoresces upon undergoing a cycloaddition.

Zinc is a vital trace element and is required in the basic cellular function of many biological organisms.^1^ In humans, proteins that bind zinc account for *ca.* 10% of all proteins,^2^ and zinc is the only transition metal found across all classes of enzymes.^3^ It is essential for important processes such as gene expression,^4^ neurotransmission,^5^ regulation of apoptosis^6^ and in reproduction.^7^ Maintenance of the correct intracellular levels of zinc is clearly requisite for critical cell functions. Indeed, misregulation of zinc in the brain is implicated in a range of neurological disorders such as epilepsy and Alzheimer's disease.^4^ Abnormal zinc levels are also intrinsically linked to cancer and are an important factor in apoptosis of malignant tumours.^8^ These factors have driven research into treatments or diagnostic tools for Zn(ii), through the design of complexation agents and sensors. Molecules which modulate their fluorescence upon undergoing a reaction have the potential to selectively indicate the location of specific functional groups or analyte, for example azide- or alkyne-containing proteins or other synthetic molecules.^9^ Azido coumarin 1 is an example of a reagent which increases significantly in fluorescence upon cycloaddition with an alkyne.^9*a*^ In cases where the partner is a strained alkyne,^10^ the cycloaddition reaction can take place without the need for a copper catalyst,^11^ which is highly advantageous in a biological setting due to its bioorthogonality (Scheme 1).^11^

**Fig. 1:**
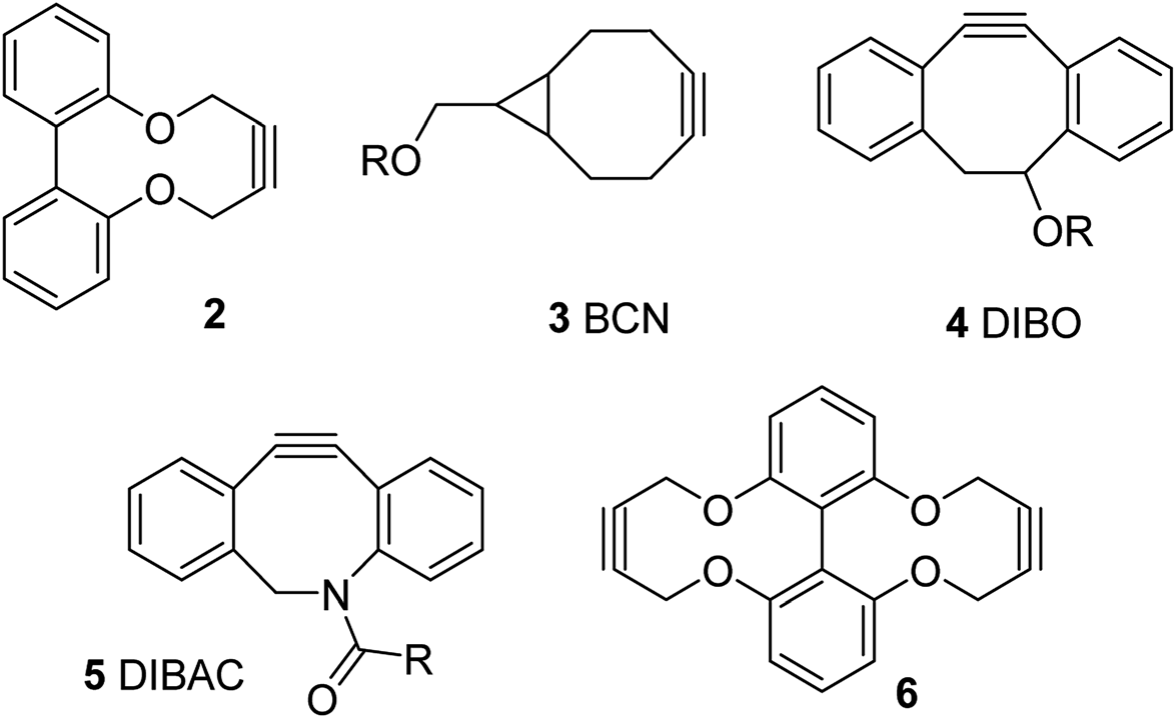
Reported strained alkynes.

We, and others, have recently reported the synthesis and reactions of a strained alkyne of general structure 2, derived from a biphenol backbone (Fig. 1).^12^ Although not as reactive as the more strained commercial reagents such as 3–5,^10,11^ it is simple to prepare *via* a short sequence, reacts with azides without a metal catalyst and has been demonstrated to be capable of forming synthetically-useful derivatives. We have also reported a dialkyne version of this reagent, *i.e.*6.^12*d*^

Although strained alkynes such as 3–5 can be functionalised with a fluorescent group,^13^ we felt that it would be advantageous to design a reagent which could potentially gain fluorescence upon undergoing the cycloaddition reaction. Toward this end we designed reagent 7, which contains a bipyridyl functional group, reasoning that its out-of-plane locking of the aryl rings by the inflexible alkyne would prevent fluorescence and eliminate the potential for metal chelation between the pyridiyl nitrogen atoms. However, upon cycloaddition with an azide, the rotation barrier about the biaryl C–C bond should be decreased, increasing the propensity to become co-planar and thus become weakly emissive, which has the potential to increase further upon chelation of a suitable metal ion such as Zn(ii), which are known to be highly emissive.^14^ Related reagents based on the same dipyridyl scaffold have been reported,^15^ providing some precedent for our synthetic approach.

## Results and discussion

**Scheme 2:**
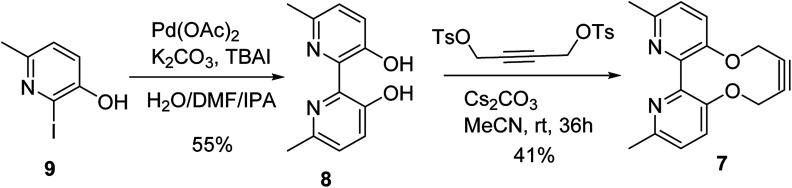
Synthesis of 2,2-bipyridyl strained alkyne 7.

The target reagent, 7, was prepared through a two-step process by first forming the known 2,2′-bipyridyl-3,3′-diol 8 ^16^ from iodopyridine 9 using a new protocol, and then cyclising the reagent through reaction with but-2-yne-1,4-diyl bis(4-methylbenzenesulfonate), following our established protocol, to give 7 in 41% yield. The ^1^H NMR spectrum of 7 exhibited diastereotopic CH_2_ resonances indicating conversion between atropisomers is slow on the NMR timescale (500 MHz, CDCl_3_, 298 K). Correspondingly, it was found that compound 7 was not an effective bidentate ligand, likely due to the large C–C biaryl torsional angle; our previous solid-state studies of biphenol strained alkynes evidence significantly non-planar C–C biaryl bond dihedral angles (Scheme 2).^12*a*^

**Scheme 3:**
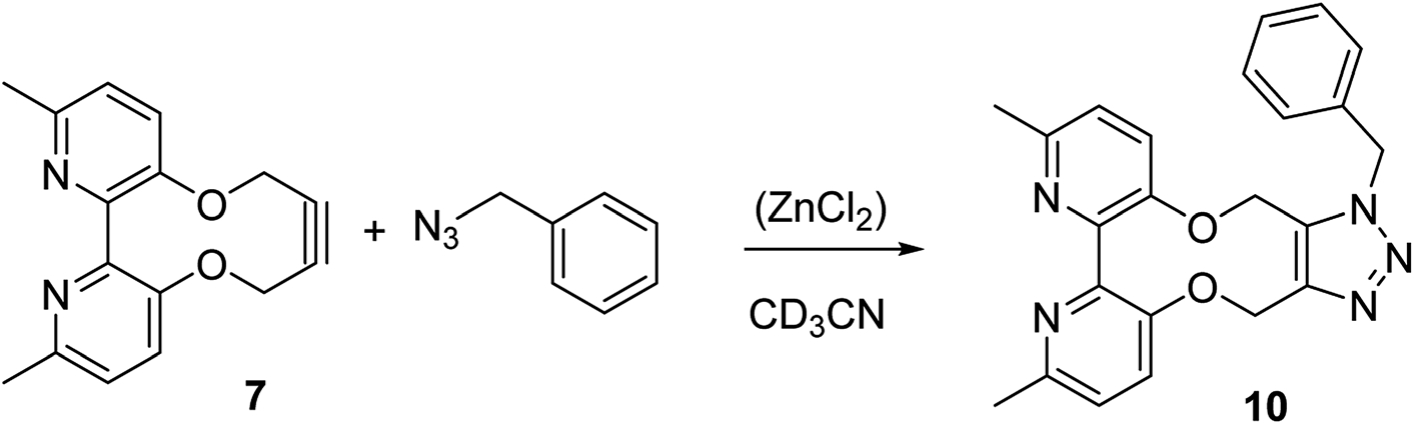
Cycloaddition of bipyridyl strained alkyne 7 with benzyl azide. The reaction was run with and without ZnCl_2_ in order to gauge its effect on the rate.

The cycloaddition of 7 with benzylazide was studied (Scheme 3), using ^1^H-NMR to follow the changes *in situ*. The reaction took place without the requirement for a copper catalyst, generating the cycloadduct 10 in high yield over several days. The adduct 10 was qualitatively more fluorescent than the 2,2′-bipyridyl strained alkyne 7, presumably due to the reduced rotational restriction upon cycloaddition. The cycloaddition in the presence of ZnCl_2_ was slightly faster than in its absence and gave a significantly emissive adduct. It is unclear as to how the Zn(ii) accelerates the reaction however it is unlikely to chelate to the two pyridyl rings due to the likely wide torsion angle in the strained alkyne (*vide supra*).

**Fig. 2:**
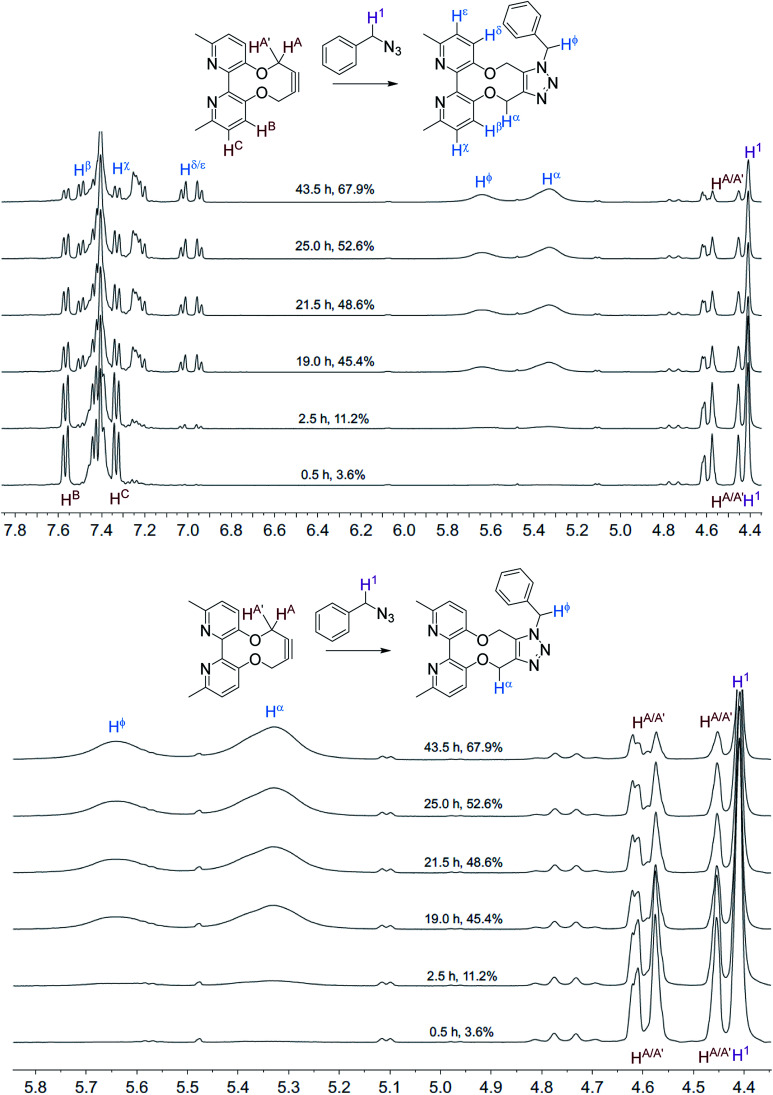
Overlaid ^1^H NMR spectra (400 MHz, CD_3_CN, 298 K) for the cycloaddition reaction in Scheme 3 without added ZnCl_2_.

The NMR spectra, recorded at time intervals, of the cycloaddition without ZnCl_2_ are overlaid in Fig. 2; the product CH_2_ resonances are broad, indicating that the detected hydrogen atoms are alternating between different conformations due to restricted rotations on the NMR time scale, as previously observed for cycloadducts of this type.^12^ A variable temperature ^1^H NMR study of 10 (500 MHz, CDCl_3_) revealed that the slow exchange of the atropisomers is reached at 243 K, contrasting 7 which is resolved at ambient temperature, indicating greater flexibility of 10, however coalescence was not reached at 323 K (ESI†).

**Fig. 3:**
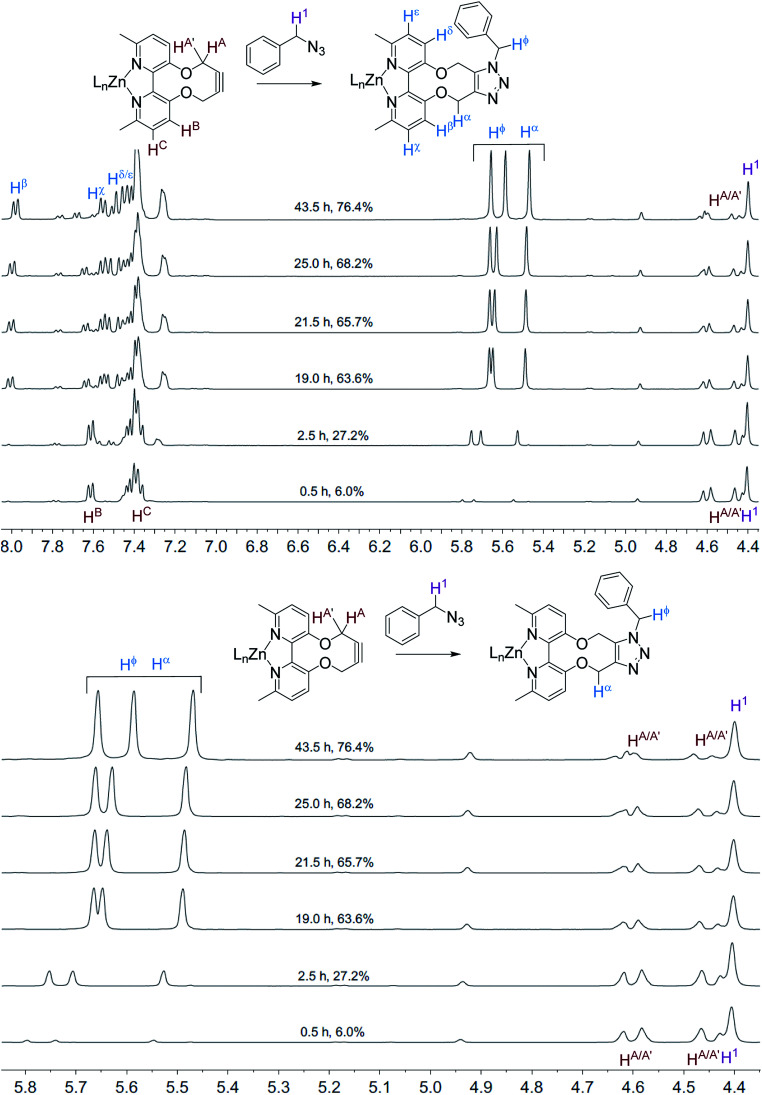
Overlaid ^1^H NMR spectra (400 MHz, CD_3_CN, 298 K) for the cycloaddition reaction in Scheme 3 with added ZnCl_2_.

**Fig. 4:**
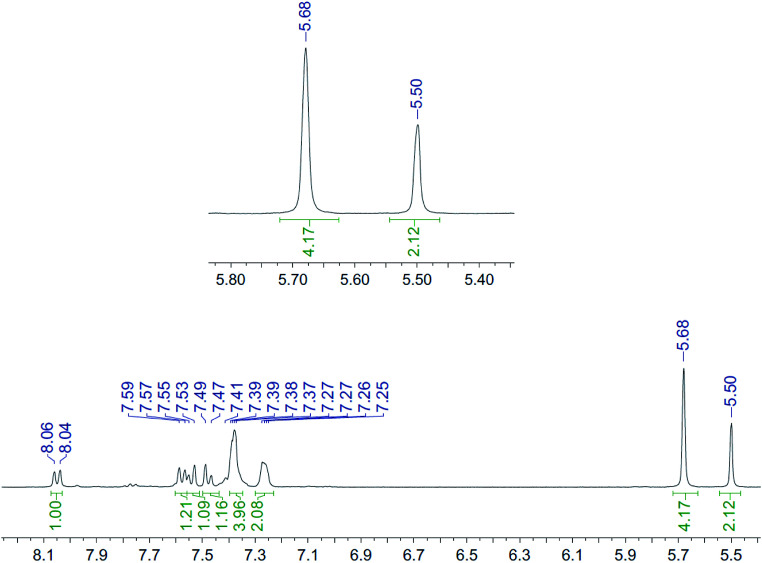
^1^H NMR spectrum (400 MHz, CD_3_CN, 298 K) for the uncomplexed addition product 10 after adding ZnCl_2_.

The NMR spectra recorded, at each time interval, of the ZnCl_2_-complexed cycloaddition reaction are similarly overlaid in Fig. 3. In this case the product peaks were sharp, with three singlets being formed, corresponding to each of the CH_2_ groups in the product. In an independent test, ZnCl_2_ (1.0 eq., CD_3_CN) was added to the uncomplexed NMR sample, resulting in sharp NMR peaks similar to the Zn(ii)-complexed product 10 (Fig. 4).

**Fig. 5:**
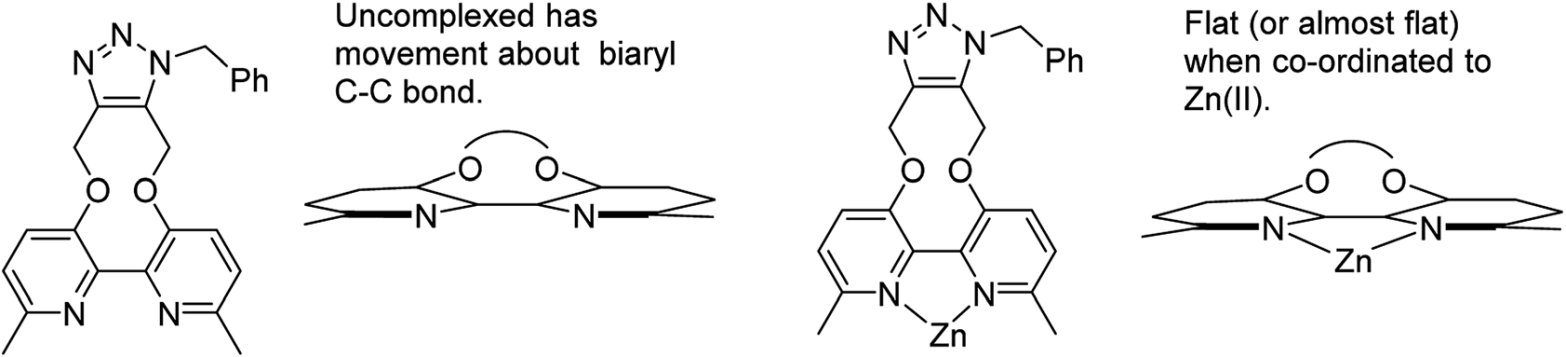
Structures of uncomplexed and Zn(ii)-complexed bipyridyl cycloaddition product 10.

It is unclear why the compound which had Zn(ii) added before the cycloaddition reaction has three singlet peaks that each integrate to two hydrogens, while the compound which had Zn(ii) added after the cycloaddition reaction has two singlet peaks, one of which integrates to four hydrogens and the other which integrates to two hydrogens. Since Zn(ii) is able to form complexes with up to three bipyridyl compounds, this could be due to a different ratio of Zn(ii) to bipyridyl in each sample.^14^ The observations indicate that the Zn(ii) complexation is likely to be responsible for the change in appearance of the NMR spectrum, possibly due to the flattening of the ring and reduction of the degrees of freedom (Fig. 5).

**Fig. 6:**
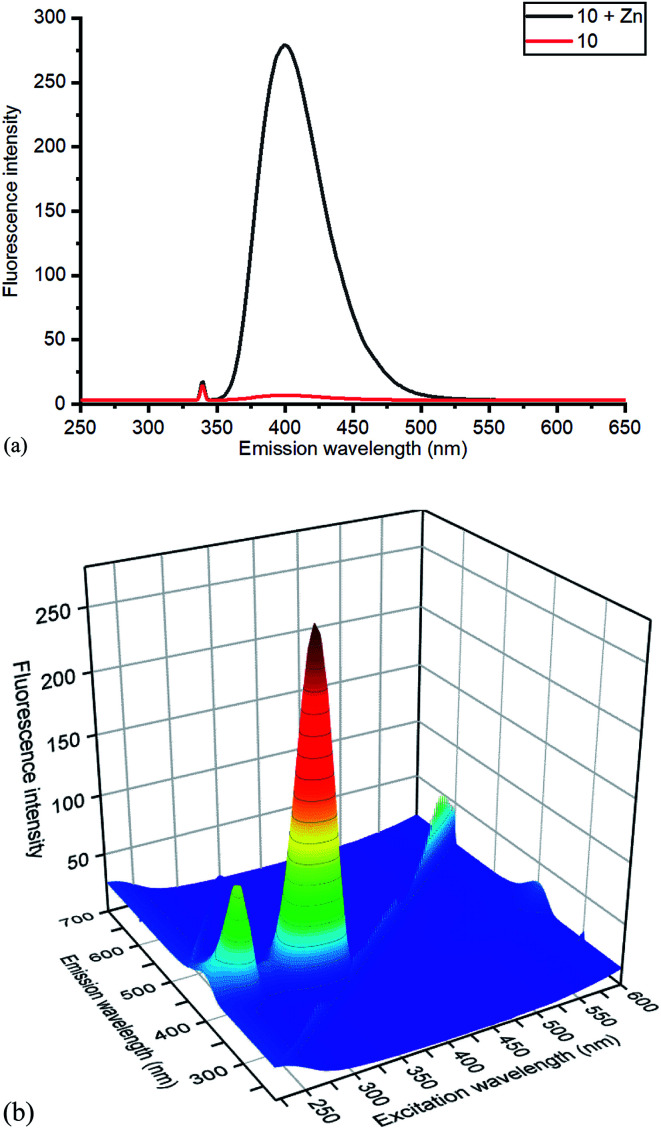
(a) Emission spectra of 10 without and with added ZnCl_2_, with excitation at 340 nm, and (b) 3D excitation *vs.* emission spectrum of 10/ZnCl_2_. The 3D spectrum of uncomplexed 10 is given in the ESI.†

Whilst this Zn(ii) complexation with a bipyridyl strained alkyne cycloaddition adduct is novel, Zn(ii) complexation with bipyridyl compounds, to create highly fluorescent adducts, has been previously studied in detail.^14^ Zn(ii) complexation has been shown to increase fluorescence intensity, a property which could potentially be beneficial for fluorescence-based reagents. The effect of Zn(ii) complexation on fluorescence was also observed on both the cycloaddition bipyridyl product 10 and the precursor bipyridyl diol 8. All samples were made to the same concentration (10^−5^ M), and values were recorded at the same parameters on the same instrument (Fig. 6). The strained alkyne reagent 7 was also studied for comparison purposes.

**Scheme 4:**
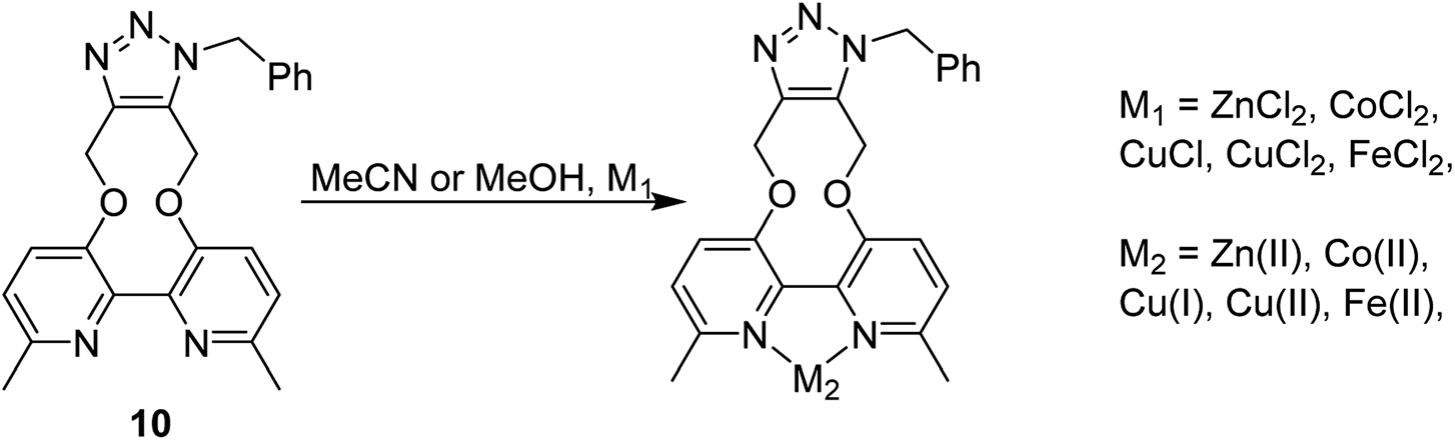
Metal complexation with bipyridyl product 10.

**Fig. 7:**
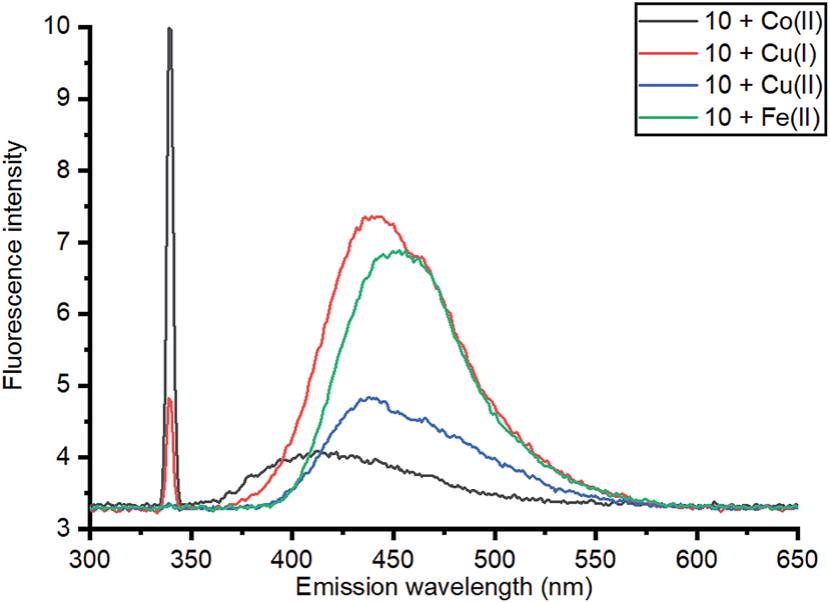
Comparison of emission of complexes of 10 with a range of metals at 340 nm excitation; 3D and contour excitation *vs.* emission spectra are given in the ESI.†

Based on relative intensity, cycloaddition product 10 showed a significant increase in fluorescence upon addition of ZnCl_2_ (Fig. 6) In contrast, addition of Zn(ii) to the solution of 7 resulted in quenching of the fluorescence (ESI†). This indicated that chelation of the metal was required for increased fluorescence, since the rigid structure of 7 prevents this. After observing the increase in fluorescence with Zn(ii) for cycloaddition bipyridyl product 10, it was complexed with several other metal salts to investigate the selectivity of the system. The 3D fluorescence spectra were recorded for each of the metal complexes (Scheme 4, Fig. 7) The 3D and contour excitation *vs.* emission spectra are given in the ESI†.

**Fig. 8:**
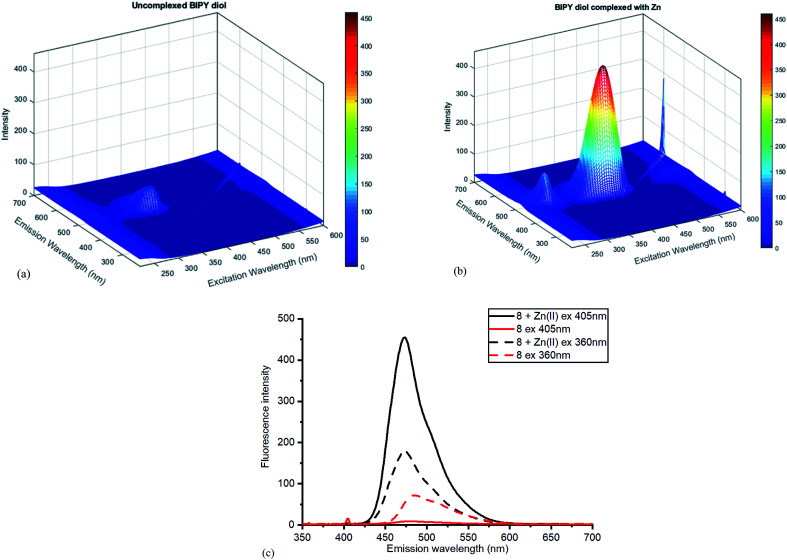
Fluorescence spectra for diol 8 (a) without added ZnCl_2_ (b) with added ZnCl_2_. (c) Contrasting emissions at 405 and 360 nm excitation with and without added Zn(ii).

The Zn(ii)-complexed bipyridyl 10 exhibited a significantly higher fluorescence intensity compared to the other metal complexes; none of the other metals enhanced the fluorescence of the bipyridyl 10; moreover, the other metal complexes had lower emission intensities than even the uncomplexed bipyridyl 7. Table 1 summarises the combination of excitation and emission wavelength that gave the highest response as determined by 3D fluorescence spectroscopy, and the Stokes shifts. For comparison, the fluorescence of the ZnCl_2_-complexed and uncomplexed bipyridyl diol precursor 8 ^17^ was also measured using 3D fluorescence spectroscopy (Fig. 8).

**Table 1:** Intensities at the maximum excitation and emission wavelengths for metal complexes of 10^a^

	Excitation *λ*_max_ (nm)	Emission *λ*_max_ (nm)	Intensity	Shift/nm
Uncomplexed 10	295	379	23	85
10 Zn II complex	341	400	279	59
10 Cu I complex	340	439	7	99
10 Cu II complex	345	438	5	93
10 Co II complex	325	450	6	75
10 Fe II complex	335	450	8	115

a Spectra were recorded at a concentration of 10^−5^ M in MeCN.

Once again, the Zn(ii) complexation caused a dramatic increase in the fluorescent intensity of the compound, although the selection of excitation wavelength was crucial in establishing the sharpest difference between the complexed and uncomplexed emission. With excitation at 405 nm, sharply contrasting emission results were obtained compared to those at 360 nm excitation, where little difference was observed (Fig. 8c). The high fluorescence reflects efficient chelation of the Zn(ii) by diol 8.

## Conclusion

In conclusion, we have prepared a new strained alkyne based on a bipyridyl dioxy backbone and have applied this to strain-promoted cycloaddition reactions with benzyl azide. Metal complexation is precluded on the alkyne compound 7 due to the rigidity and non-coplanarity of the bipyridine. Upon triazole formation, product 10 increases in conformational flexibility, allowing coordination to a range of transition and alkaline earth metals, as evidence by photophysical studies. Importantly, the fluorescence intensity increases significantly when Zn(ii) salts are added to it, which is selective across the ranges of metals studied.

## Data sharing statement

The research data (and/or materials) supporting this publication can be accessed at http://wrap.warwick.ac.uk/.

## Conflicts of interest

The authors declare no conflicting interests.

## Supplementary Material

RA-009-C9RA06866J-s001
