# Supplementary material for: A strained alkyne-containing bipyridine reagent; synthesis, reactivity and fluorescence properties
Source: RSC Adv. 2019 Nov 6;9(62):36154–61. doi: 10.1039/c9ra06866j (PMC9074932; doi:10.1039/c9ra06866j)

Electronic Supplementary Material (ESI) for RSC Advances.
This journal is © The Royal Society of Chemistry 2019

Supporting information.

A strained alkyne-containing bipyridine; synthesis, reactivity and fluorescence properties.

Sam Forshaw,[a] Richard C. Knighton,[a] Jami Reber,[a] Jeremy S. Parker,[b] Nikola Chmel[a] and Martin Wills.[a*]

a. Department of Chemistry, The University of Warwick, Coventry, CV4 7AL, UK.

b. Early Chemical Development, Pharmaceutical Sciences, IMED Biotech Unit, AstraZeneca, Macclesfield, SK10 2NA, UK.

**Experimental Details**	**S2**
**Effect of addition of Zn(II) on cycloaddition rate**	**S10**
**Fluorescence Spectroscopy of cycloaddition bipyridyl product 10, precursor 7 and diol 8**	**S15**

Experimental section:

General Experimental.

All reagents and solvents were used as purchased and without further purification. All reactions were carried out under a nitrogen atmosphere unless otherwise specified. Reactions at elevated temperature were maintained by thermostatically controlled aluminum heating blocks. A temperature of 0 °C refers to an ice slush bath. Standard NMR spectra were recorded on a Bruker DPX (300 or 400 MHz), or Bruker DRX (500 MHz) spectrometer at 298 K unless otherwise stated. Variable temperature NMR experiments were performed on a Bruker Avance (500 MHz) spectrometer. All chemical shifts are reported in ppm and are referenced to the solvent chemical shift, and coupling constants are given in Hz. Mass spectra were recorded on an Esquire 2000 and high resolution mass spectra were recorded on a Bruker Micro ToF or MaXis. IR spectra were recorded on a PerkinElmer spectrum100 and peaks are reported in wavenumbers. The optical rotations were measured on an Optical Activity Ltd. AA-1000. Flash column chromatography was performed using silica gel of mesh size 230-400, Thin layer chromatography was carried out on aluminum backed silica gel 60 (F254) plates, visualised using 254 nm UV light or potassium permanganate stains as appropriate. Fluorescence spectra were recorded on a Jasco FP-6500 spectrofluorometer and maximum excitation and emission wavelengths are reported in nm.

2-Iodo-6-methylpyridin-3-ol 9.

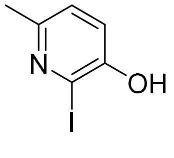

This compound is known and fully characterised.

Iodine (5.2 g, 20 mmol, 1.1 eq.) and $NHCO_3$ (1.68 g, 20 mmol, 1.1 eq.) were added to a stirring solution of 2-methyl-5-hydroxypyridine (2.0 g, 18 mmol, 1.0 eq.) in a 1:1 mixture of THF/H_2O (30 mL). The black reaction mixture was stirred for 48 hours before a 5 % solution of $Na_2S_2O_3$ was added dropwise until the mixture became colourless and a white precipitate formed. The precipitate was removed by filtration and dried in a desiccator *in vacuo* to give the product **9** as a white solid (3.14 g, 13.4 mmol, 74%).

δ_H (300 MHz, $CDCl_3$) 7.11 (1H, d, *J* 8.0, ArH), 6.98 (1H, d, *J* 8.0 , ArH) and 2.47 (3H, s, CH_3);

δ_C (101 MHz, $CDCl_3$) 152.5, 150.4, 124.1, 122.0, 110.3 and 23.2;

m/z (ESI) 234. 0 (M-H$^-$, 100%).

The data matched that reported for this compound; Anna Reynal, Juan Etxebarria, Natalia Nieto, Sílvia Serres, Emilio Palomares, Anton Vidal-Ferran, *Eur. J. Inorg. Chem.*, 2010, 1360-1365.

^{1}**H NMR** spectrum (500 MHz, $CDCl_3$)

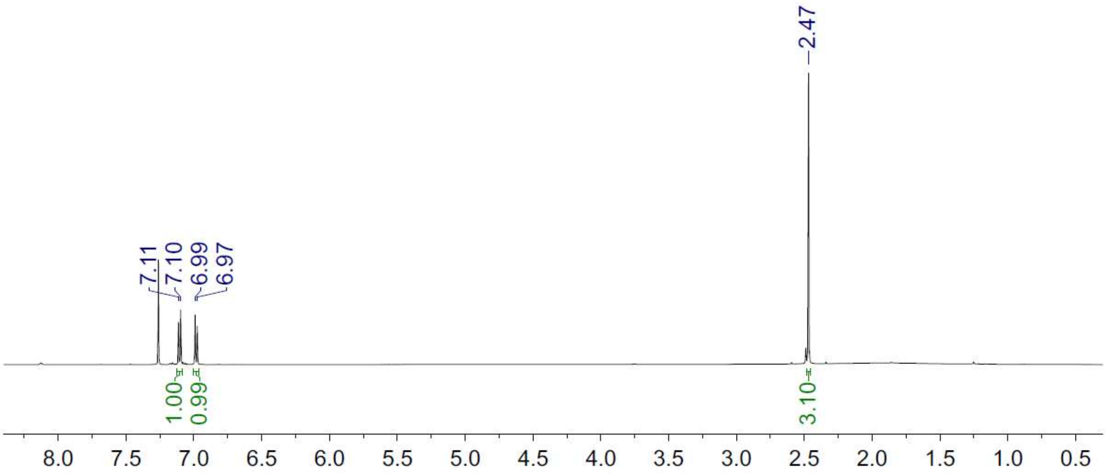

^{13}**C NMR** spectrum (126 MHz, $CDCl_3$)

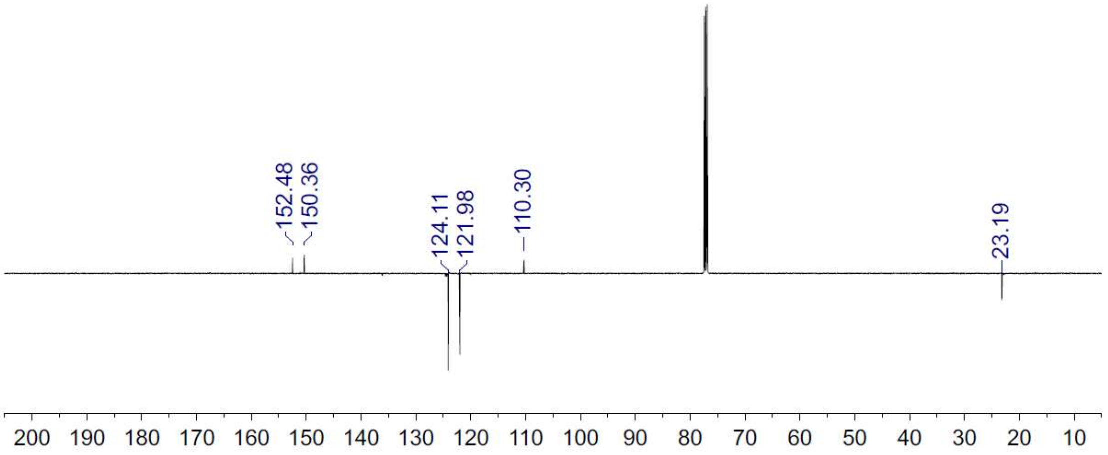

3,3’-Dihydroxy-6,6’-dimethyl-2,2’-bipyridine 8.

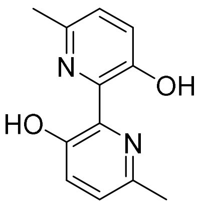

This compound is known and fully characterised. .

After stirring a mixture of 2-iodo-6-methylpyridin-3-ol **9** (2.00 g, 8.50 mmol, 1.0 eq.), TBAI (1.57 g, 4.25 mmol, 0.5 eq.), K_2CO_3 (1.29 g, 9.35 mmol, 1.1 eq.) and $Pd(OAc)_2$ (95 mg, 0.425 mmol, 0.05 eq.) in 1:3 H_2O/DMF (1.6 mL) at 115 °C for 10 minutes, IPA (0.65 mL, 510 mg, 8.5 mmol, 1.0 eq.) was added and the mixture stirred at 115 °C for an additional 48 hours. The mixture was cooled to room temperature and diluted with EtOAc (20 mL). The organic layer was then washed with H_2O (3 × 20 mL) and separated and the combined aqueous layers were extracted with EtOAc (3 × 20 mL). The combined organic layers were then dried over $MgSO_4$ and the solvent removed *in vacuo*. The product was then purified by column chromatography (SiO_2; CH_2Cl_2), to give the pure product **8** as a yellow solid (501.3 mg, 2.32 mmol, 55%).

δ_H (400 MHz, $CDCl_3$) 14.73 (2H, s, 2 × OH), 7.30 (2 H, d, *J* 8.3, ArH), 7.10 (2H, d, *J* 8.3, ArH) and 2.52 (6H, s, 2 × CH_3);

δ_C (101 MHz, $CDCl_3$) 154.1, 144.8, 138.7, 126.5, 124.66, 22.9;

m/z (ESI) 217.2 ($M+H^+$, 100%).

The data matched that reported for this compound; Anna Reynal, Juan Etxebarria, Natalia Nieto, Sílvia Serres, Emilio Palomares, Anton Vidal-Ferran, *Eur. J. Inorg. Chem.*, 2010, 1360-1365).

^{1}H NMR spectrum (500 MHz, $CDCl_3$)

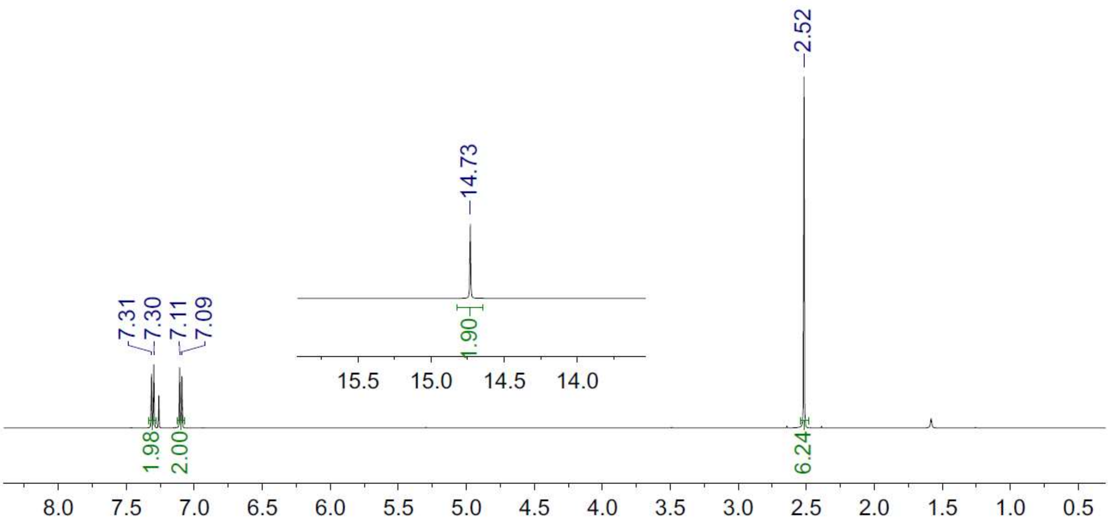

^{13}C NMR spectrum (126 MHz, $CDCl_3$)

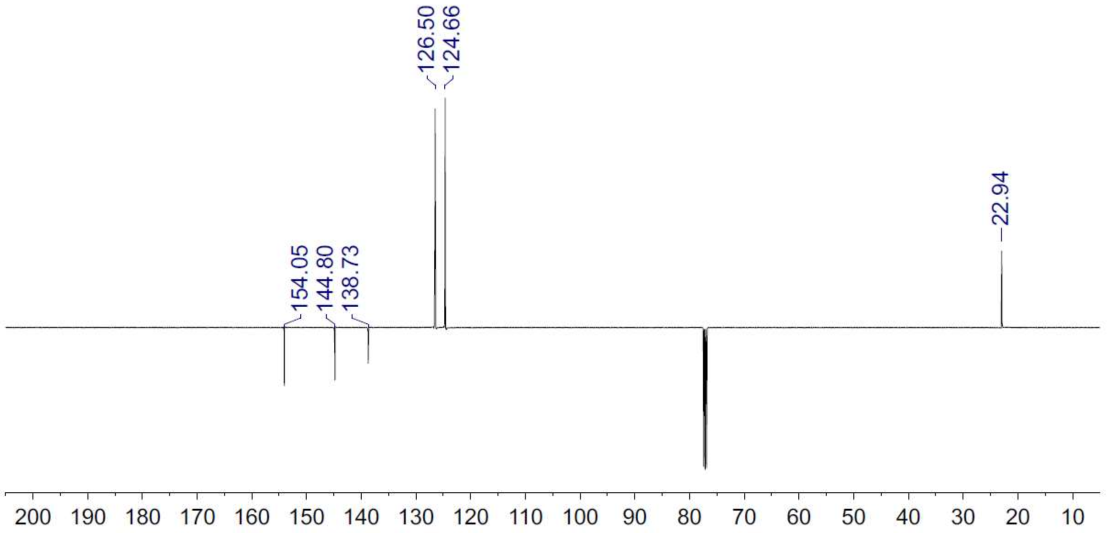

6,6'-Dimethylbipyridine alkyne 7.

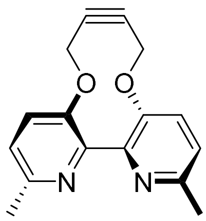

This compound is novel.

But-2-yne-1,4-diyl bis(4-methylbenzenesulfonate) (1.09 g, 2.77 mmol) was dissolved in anhydrous CH_3CN (20 mL) and transferred to a syringe. 3,3'-dihydroxy-6,6'-dimethyl-2,2'-bipyridine **8** (600 mg, 2.77 mmol) was dissolved in anhydrous DMF and transferred to a

syringe. The two solutions were added via syringe pump over 8 hours to a solution of Cs_2CO_3 (1.99 g, 6.10 mmol) in CH_3CN (20 mL) under N_2. The reaction was stirred at room temperature for 36 hours. The solution was filtered and washed with EtOAc (3 × 10 mL) and the solvent removed *in vacuo*. The product was purified by column chromatography (SiO_2; CH_2Cl_2/MeOH; 100: 0→96:4) to afford compound **7** as a fluffy off white solid (306 mg, 1.15 mmol, 41%).

MP; 176.3-181.7 ºC.

δ_H (500 MHz, $CDCl_3$) 7.43 (2 H, d, *J* 8.3, ArH), 7.22 (2 H, d, *J* 8.3, ArH), 4.66 – 4.59 (2 H, m, 2H, OCH_2), 4.38 – 4.32 (2 H, m, OCH_2), 2.57 (s, 6H);

δ_C (126 MHz, $CDCl_3$) 154.0, 153.1, 149.5, 131.3, 124.40, 87.0, 63.7, 24.2;

(found (ESI) $[M + H]^+$, 267.1133. $C_{16}H_{15}N_2O_2$ requires $[M + H]^+$ 267.1128).

m/*z* (ESI) 267 (M^+ + H, 100%) and 289 (M^+ + Na, 45);

ν_{max} 1573, 1436, 1171, 965, 953 and 725 cm^{-1};

Mp 88-92 °C;

UV-Vis (MeCN) lmax (ε/M^{-1} cm^{-1}): 279 (10,600) nm;

Fluorescence (MeCN; λ_{ex} = 300 nm); λ_{em} 368 nm;

^{1}H NMR spectrum (500 MHz, $CDCl_3$)

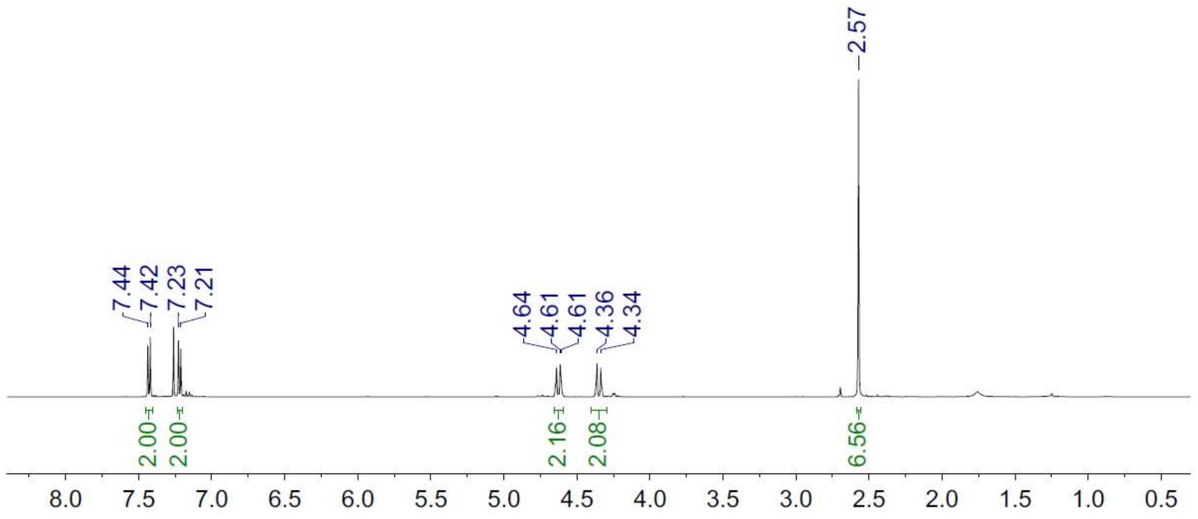

^{13}**C NMR** spectrum (126 MHz, $CDCl_3$)

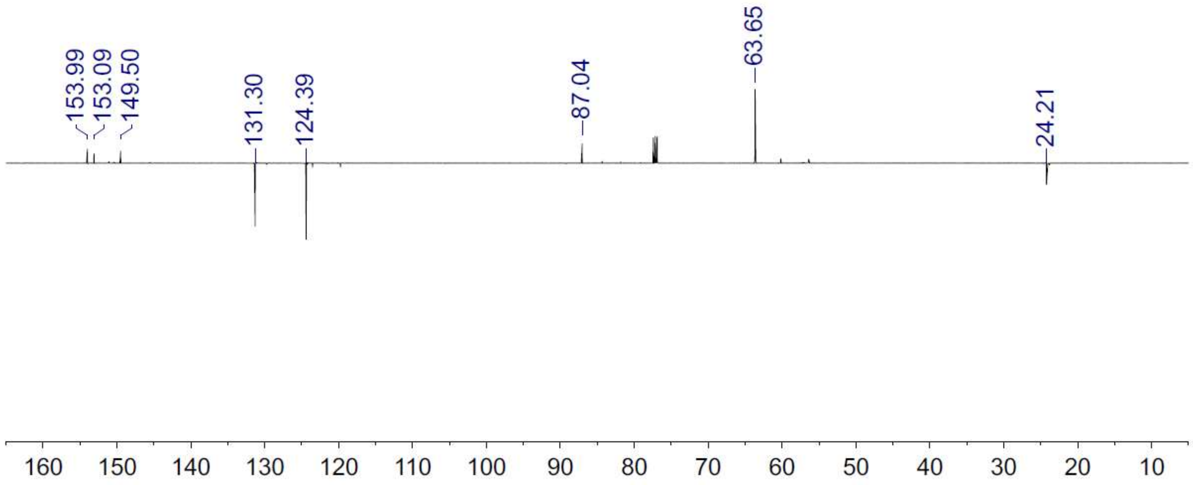

6,6'-Dimethylbipyridine triazole cycloaddition product 10.

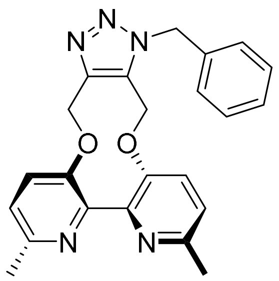

This compound is novel.

6,6'-Dimethyl-2,2'-bipyridine alkyne **7** (100 mg, 0.38 mmol) and benzyl azide (56 µL, 0.38 mmol) were dissolved in $CHCl_3$ and heated at 60 °C under N_2 for 36 hours. The solvent was removed *in vacuo* and the purified by column chromatography (SiO_2; CH_2Cl_2/MeOH; 100:0→95:5) to afford compound **10** as a fluffy white solid (141 mg, 0.35 mmol, 94%).

MP; 133.0-136.7 ºC.

δ_H (500 MHz, $CDCl_3$) 7.40 – 7.30 (4 H, m, ArH and pyH), 7.18 – 7.13 (2 H, m, ArH), 7.08 (1 H, d, *J* 8.5, pyH), 6.95 (1 H, d, *J* 8.3, pyH), 6.81 (1 H, d, *J* 8.4, pyH), 5.72 (2 H, br. s, OCH_2), 5.37 (2 H, s, $ArCH_2$), 5.10 (2 H, br. m, OCH_2), 2.54 (3 H, s, $pyCH_3$), 2.52 (3 H, s, $pyCH_3$);

δ_C (126 MHz, $CDCl_3$) ^{13}C NMR (126 MHz, $CDCl_3$) δ 153.6, 152.3, 151.5, 150.5, 147.6, 146.6, 144.3, 134.5, 131.8, 129.4, 128.8, 127.2, 123.9, 123.7, 123.7, 122.5, 63.2, 61.0, 52.6, 23.9, 23.8.

(found (ESI) $[M + H]^+$, 400.1771. $C_{23}H_{22}N_5O_2$ requires $[M + H]^+$ 400.1768);

m/z (ESI) 400 (M^+ + H, 100%) and 422 (M^+ + Na, 77);

ν_{max} 2949, 1584, 1455, 1290, 1087, 989, 824 and 719 cm^{-1};

Mp 134-137 °C;

UV-Vis (MeCN) λ_{max} (ε/M^{-1} cm^{-1}): 295 (10,700) nm;

Fluorescence (MeCN; λ_{ex} = 300 nm); λ_{em} 377 nm;

^{1}H NMR spectrum (500 MHz, $CDCl_3$)

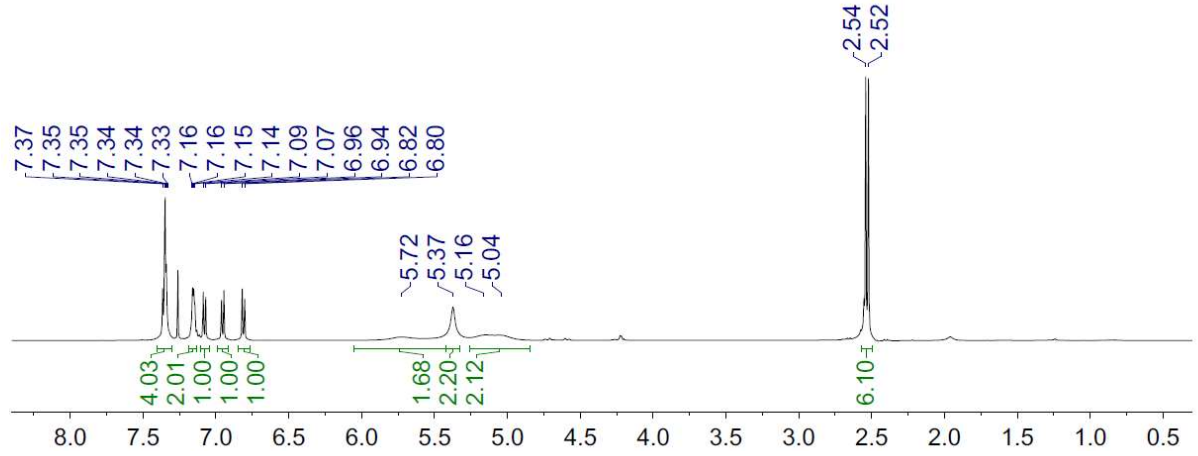

^{13}C NMR spectrum (126 MHz, $CDCl_3$)

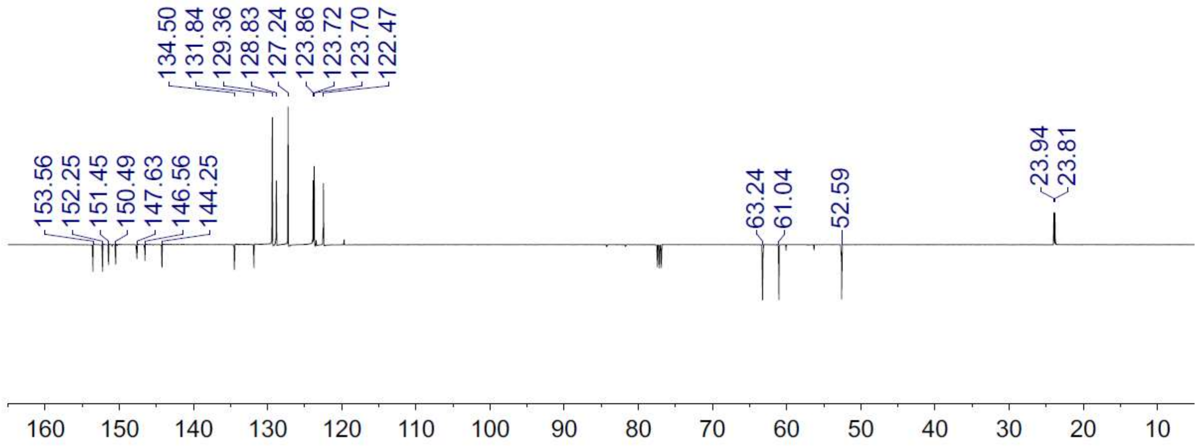

Variable temperature ^{1}H NMR spectrum (500 MHz, $CDCl_3$)

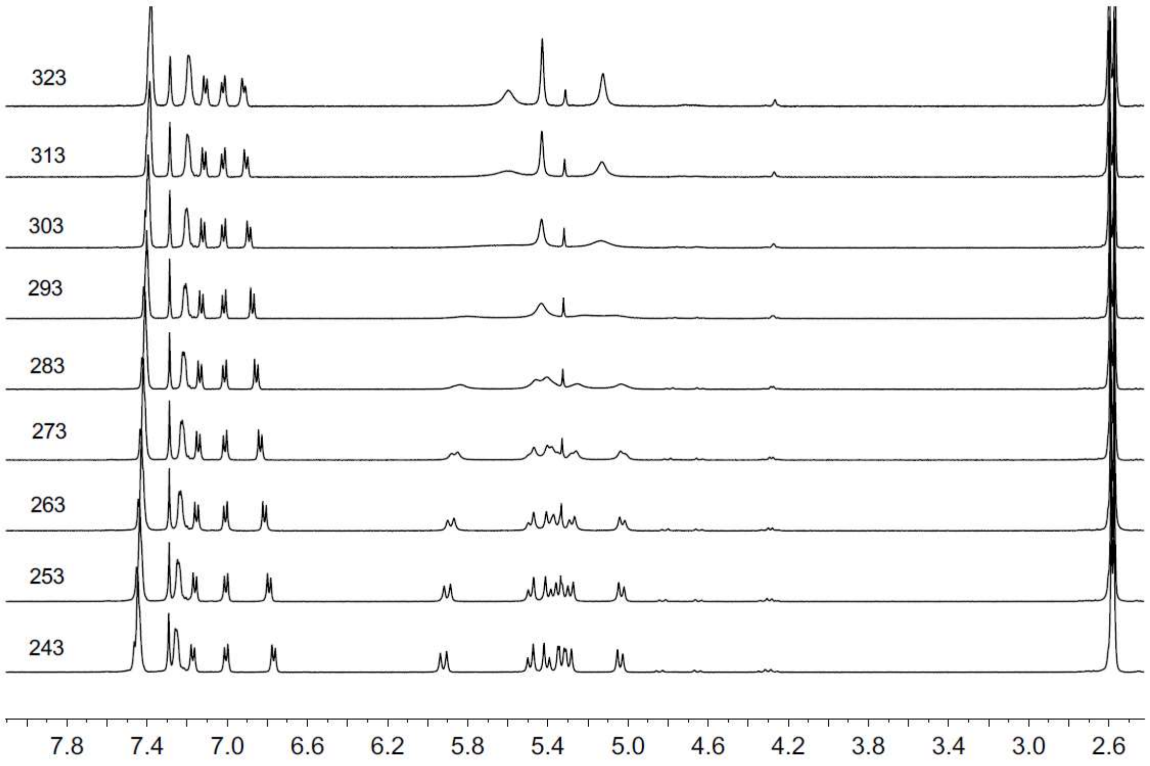

Variable temperature truncated ^{1}H NMR spectrum (500 MHz, $CDCl_3$)

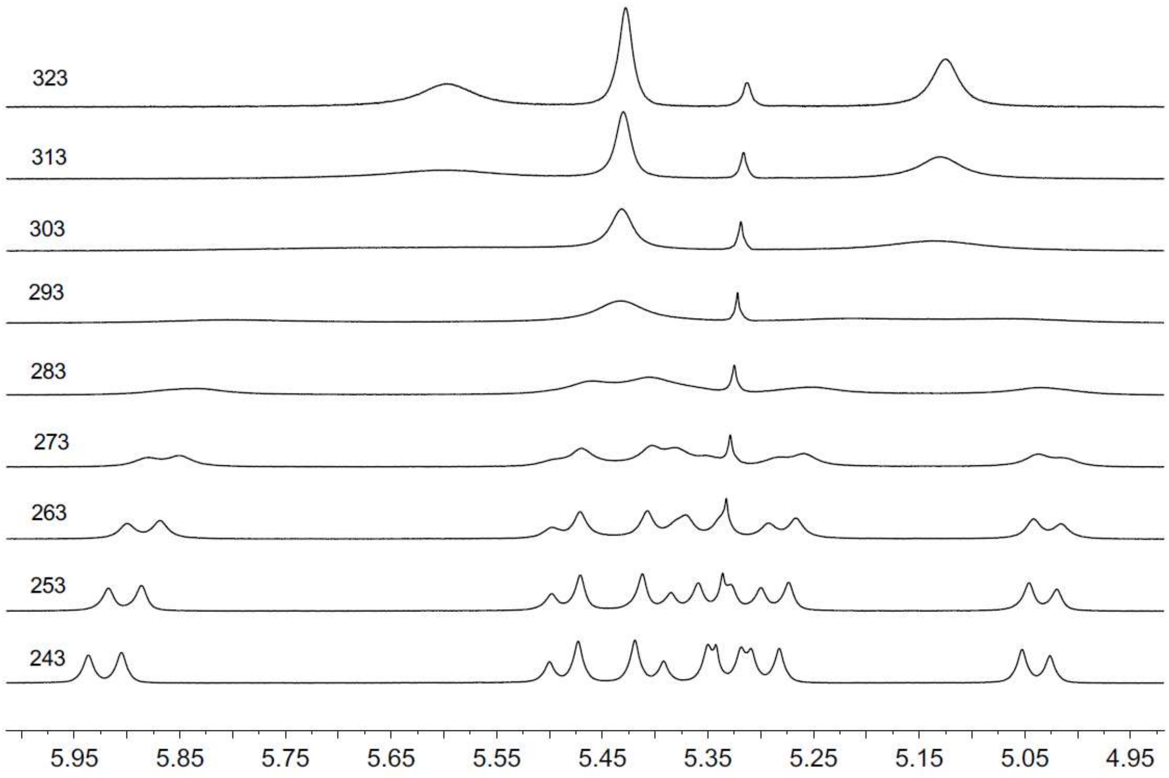

Effect of addition of Zn(II) on cycloaddition rate.

Comparison of rate of reactions of 7 with benzyl azide with and without $ZnCl_2$. (NMR featured in main paper); run in d^3-MeCN.

Two solutions of bipyridyl alkyne **9** (13.3 mg, 0.05 mmol) were dissolved in deuterated MeCN (5 mL). $ZnCl_2$ (6.81 mg, 0.05 mmol) was added to one of the solutions. Benzyl azide (6.25 μl, 0.05 mmol) was added to both solutions. Both solutions were run on ^{1}H NMR at time intervals of approximately 0 hrs, 2.5 hrs, 19 hrs, 21.5 hrs, 25 hrs, and 43.5 hrs.

(no $ZnCl_2$) δ_H (400 MHz, CD_3CN) 7.54 (1 H, d, *J* 8.6, $OCCH_A$), 7.47 (1 H, d, *J* 8.6, $OCCH_B$), 7.38 (6 H, s, 6 × ArH), 7.00 (1 H, d, *J* 8.4, $ArNCCH_A$), 6.92 (1 H, d, *J* 8.4, $ArNCCH_A$), 5.62 (2 H, s, N*H*Ph), 5.30 (4 H, 2 × OCH_2), 2.45 (6 H, s, 2 × CH_3).

(added no $ZnCl_2$) δ_H (400 MHz, CD_3CN) 7.95 (1 H, d, *J* 8.7, $OCCH_A$), 7.53 (1 H, d, *J* 8.7, $OCCH_B$), 7.36 (6 H, s, 6 × ArH), 7.24 (2 H, s, 2 × ArNCCH), 5.63 (2 H, s, OC*H*H), 5.56 (2 H, OCH*H*), 5.44 (2 H, s, N*H*Ph), 2.72 (6 H, s, 2 × CH_3).

Conversion for the uncomplexed product

Time (hrs)	Conversion (%)
0	3.66
2.5	11.2
19	45.4
21.5	48.6
25	52.6
43.5	67.9

Conversion for the Zn(II)-complexed product

Time (hrs)	Conversion (%)
0	6.02
2.5	27.2
19	63.6
21.5	65.7
25	68.2
43.5	76.4

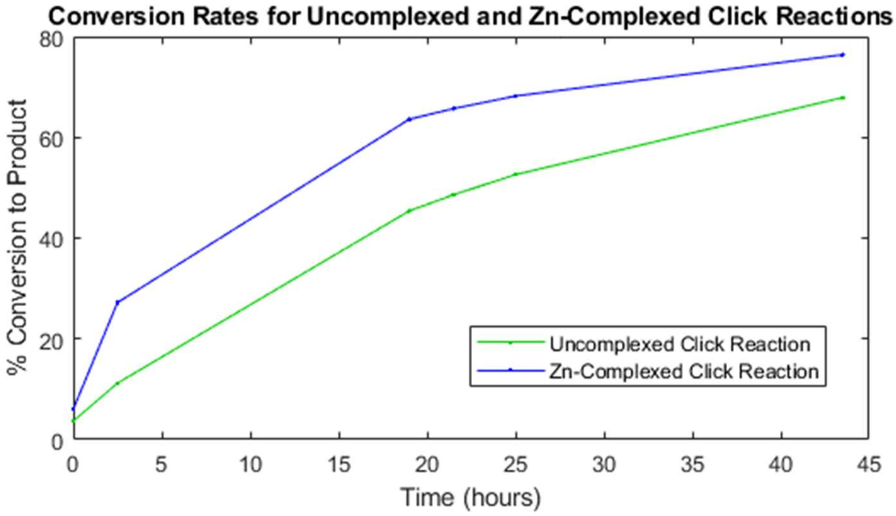

NMR spectra for addition reactions.

Reactions without added $ZnCl_2$:

^1H NMR (400 MHz, CD_3CN, 298 K)

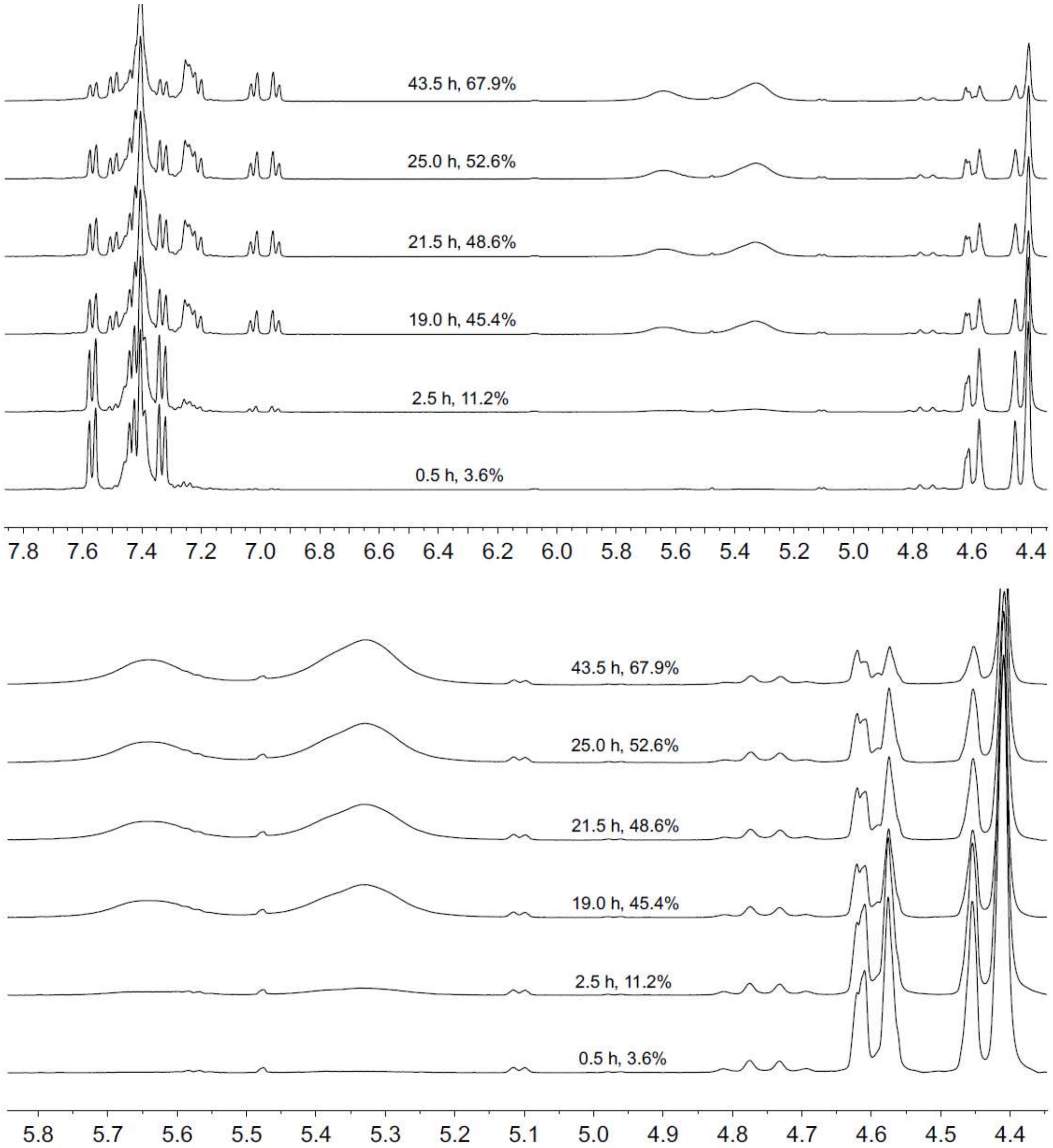

Overlaid ^1H NMR spectra (400 MHz, CD_3CN, 298 K) for the cycloaddition reaction in Scheme 3 without added $ZnCl_2$.

After adding $ZnCl_2$ to the solution.

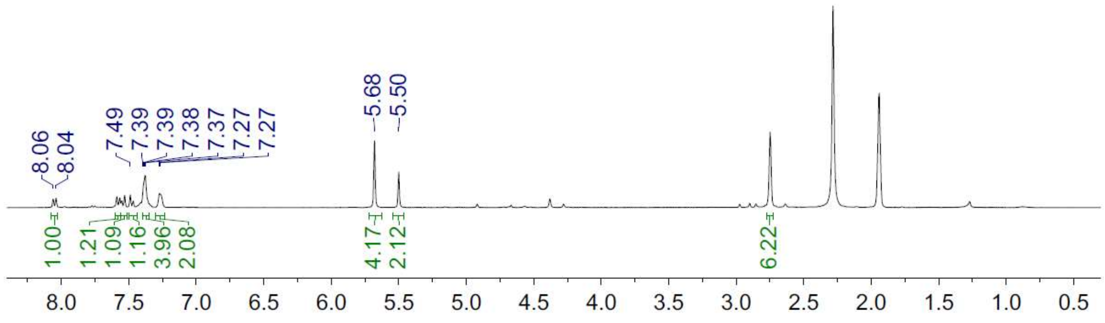

Reactions with added $ZnCl_2$:

^{1}H NMR (400 MHz, CD_3CN, 298 K)

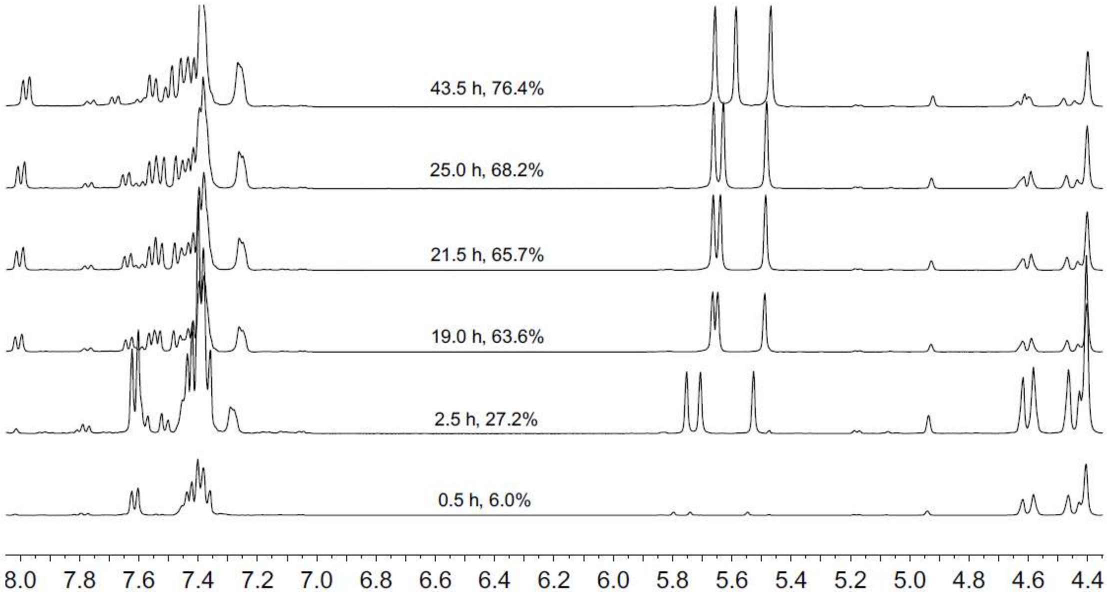

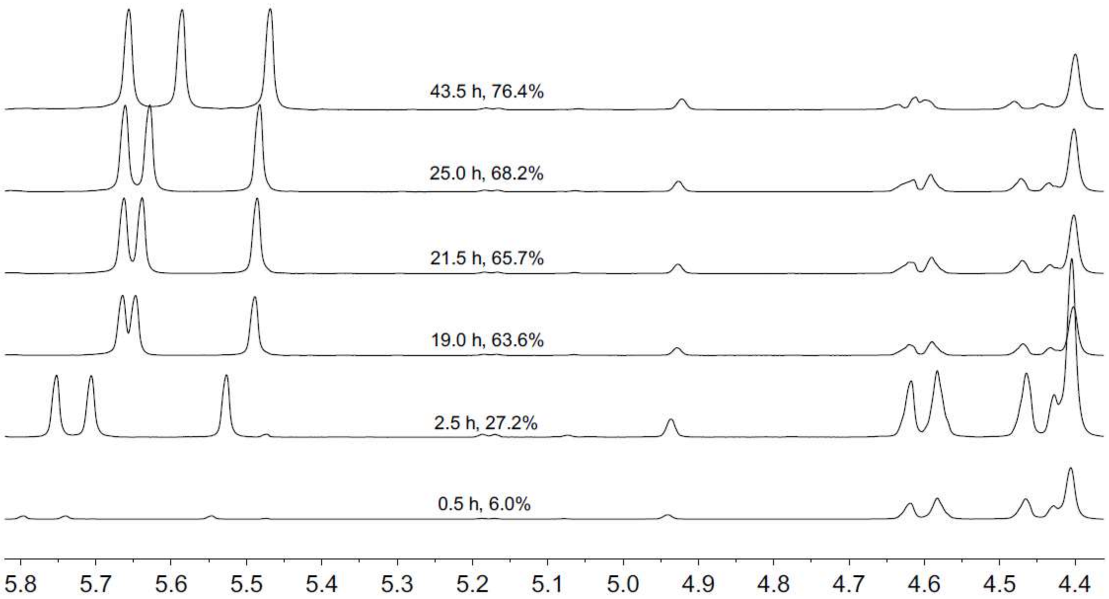

Overlaid ^{1}H NMR spectra (400 MHz, CD_3CN, 298 K) for the cycloaddition reaction in Scheme 3 with added $ZnCl_2$.

Extended runs over a longer timescale and product isolation (SF).

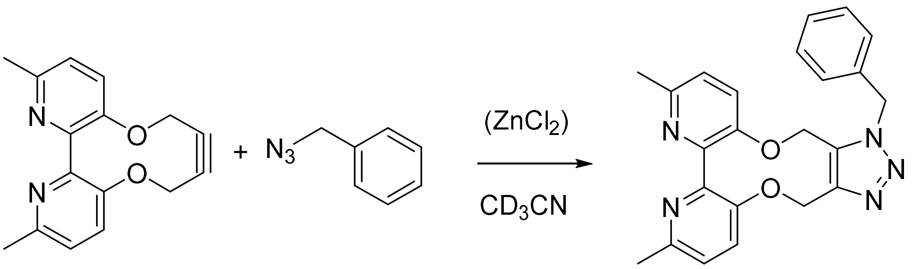

Without $ZnCl_2$: A solution of bipyridyl alkyne **7** (13.3 mg, 0.050 mmol, 1 eq) and benzyl azide (7.65 mg, 0.050 mmol, 1 eq) in *d*-MeCN (0.50 mL) was followed by ^{1}H NMR. After 6 days the solvent was removed and the product purified by flash column chromatography (SiO_2; CH_2Cl_2/MeOH; 100: 0→95:5) to give the pure product as a white solid (14 mg, 0.035 mmol, 70 %).

With $ZnCl_2$: A solution of bipyridyl alkyne **7** (13.3 mg, 0.050 mmol, 1 eq), benzyl azide (7.65 mg, 0.050 mmol, 1 eq) and $ZnCl_2$ (6.8 mg, 0.050 mmol, 1 eq) in d_3-MeCN (0.5 mL) was followed by ^{1}H NMR. After 6 days the solvent was removed *in vacuo* and the product purified by flash column chromatography (SiO_2; CH_2Cl_2/MeOH; 100: 0→95:5) to give the pure product as a white solid (14 mg, 0.035 mmol, 70 %).

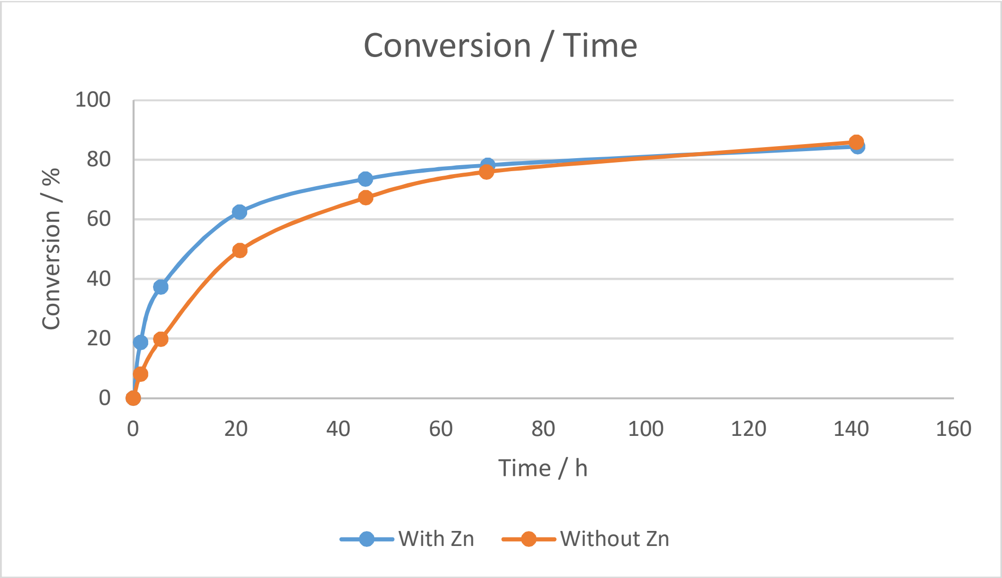
Conversion / Time
Conversion / %
100
80
60
40
20
0
0
20
40
60
80
100
120
140
160
Time / h
With Zn
Without Zn

Fluorescence Spectroscopy of cycloaddition bipyridyl product 10, precursor 7 and diol 8.

Procedure.

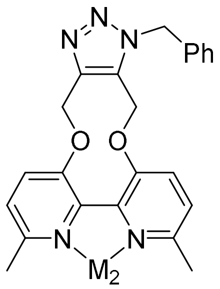

M_1 = $ZnCl_2$, $CoCl_2$, CuCl, $CuCl_2$, $FeCl_2$,

M_2 = Zn(II), Co(II), Cu(I), Cu(II), Fe(II),

A 10^{-3} M solution of cycloaddition bipyridyl product **10** (2.0 mg, 5.01 μmol) was prepared in MeCN (5 ml). This stock solution was diluted to 10^{-5} M with additional MeCN for each metal solution. Approximately 0.1 mg each of $ZnCl_2$, $CoCl_2$, CuCl, $CuCl_2$, and $FeCl_2$ were added to diluted bipyridyl solutions (5 mL, 10^{-5} M) to produce the metal-complexed solutions.

Three dimensional (3D) emission vs excitation, fluorescence spectra were obtained for each sample, and the maxima in each case are summarised in the Table in the main paper. The 3D and contour plots are presented in the data below.

Fluorescence method:

Fluorescence measurements were performed on a Jasco FP-6500 spectrofluorophotometer (Jasco, Japan) using a 1 cm path length quartz cell (Starna, UK) at ambient temperature. All 3D fluorescence measurements were performed using the following parameters: excitation wavelength range: 220-600 nm; emission wavelength range: 220-700 nm; excitation bandwidth: 1 nm; emission bandwidth: 3 nm, detector sensitivity: medium; response: 1 second; scanning speed: 100 nm/min.

UV-vis method:

UV-vis absorbance measurements were performed on a Jasco V-660 spectrometer (Jasco, Japan) using a 1 cm path length quartz cell (Starna, UK) at ambient temperature. All UV-vis absorbance measurements were performed using the following parameters: measurement range: 230-700 nm; bandwidth: 1nm; response: 1 second; scanning speed: 100 nm/min; 2 accumulations.

UV-vis spectra of (a) **7** and (b) **10** with and without Zn(II) (10^{-5} M in MeCN).

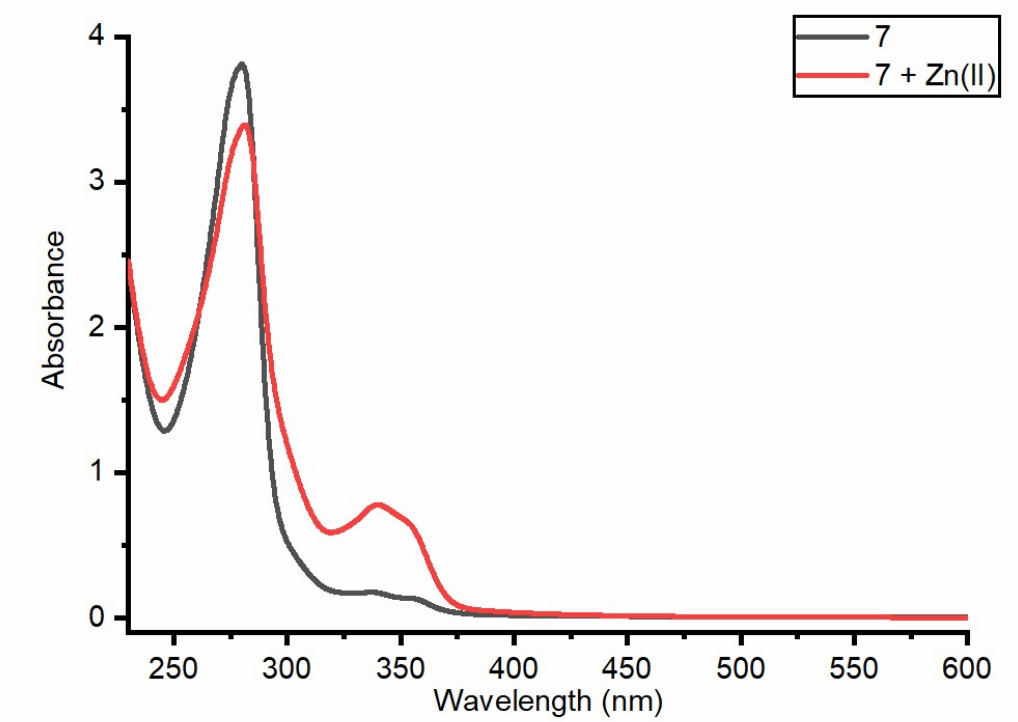

(a)

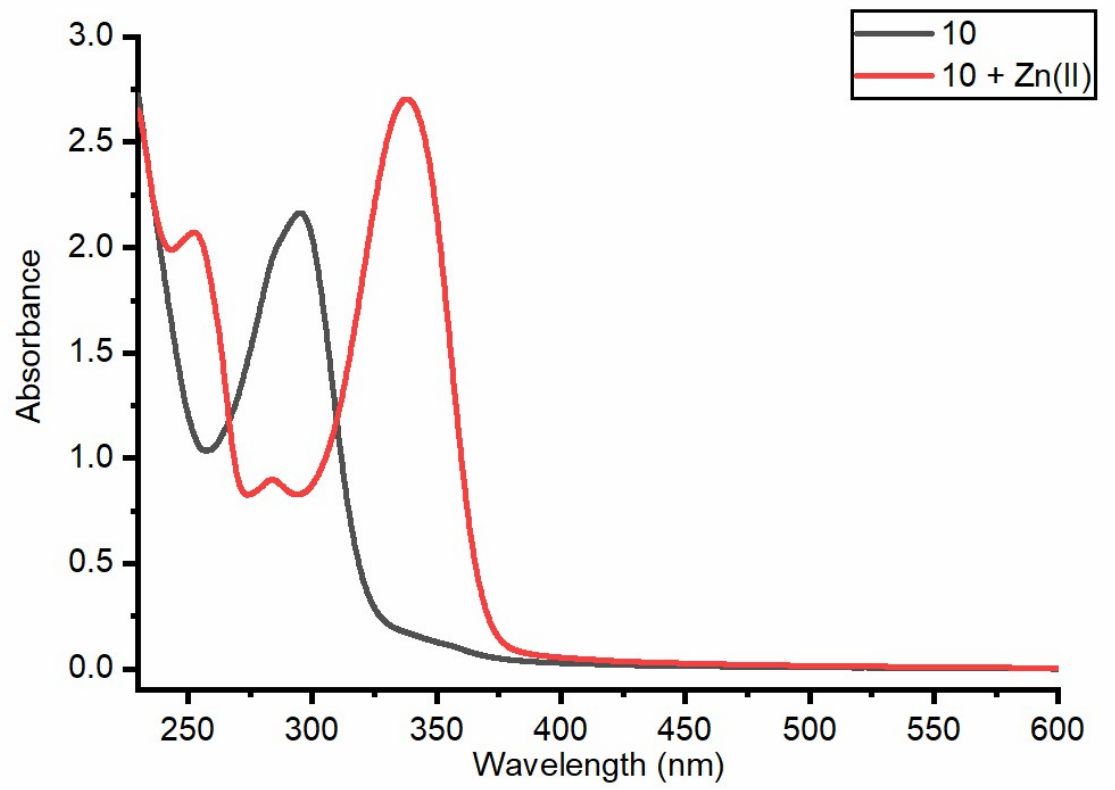

(b)

3D Excitation vs Emission Spectra of **7** (a) with and (b) without Zn(II) ((10^{-5} M in MeCN), (c) comparison of emission at excitation at 300 nm.

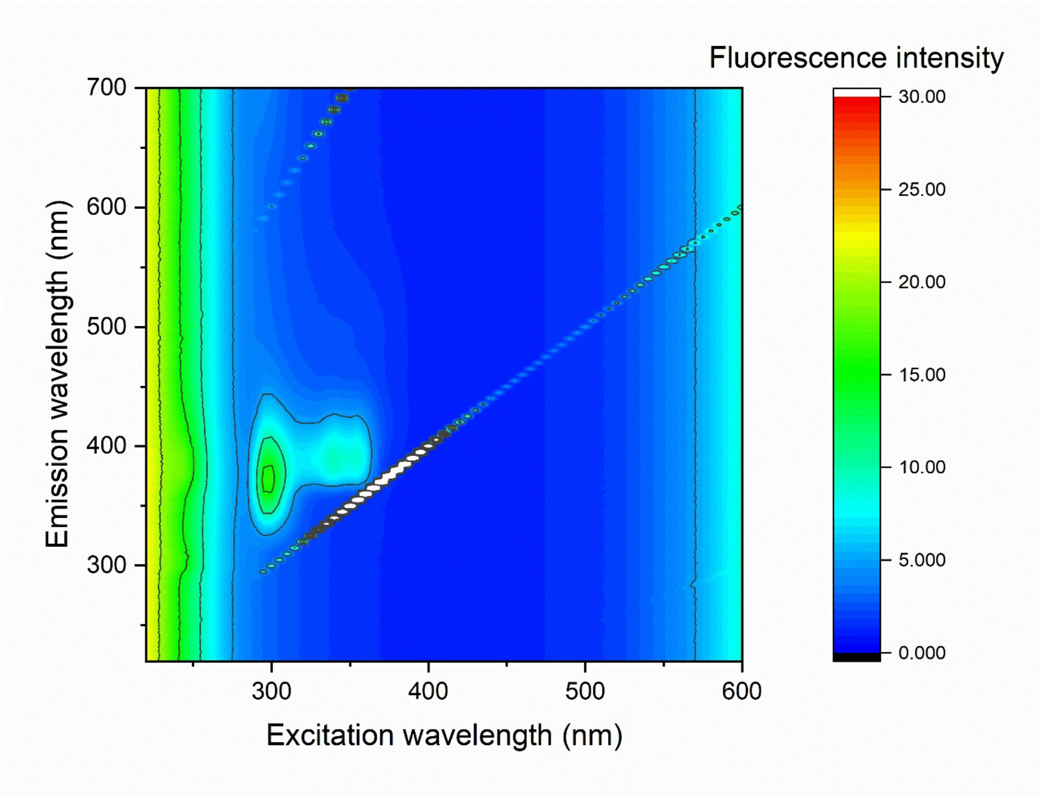

(a)

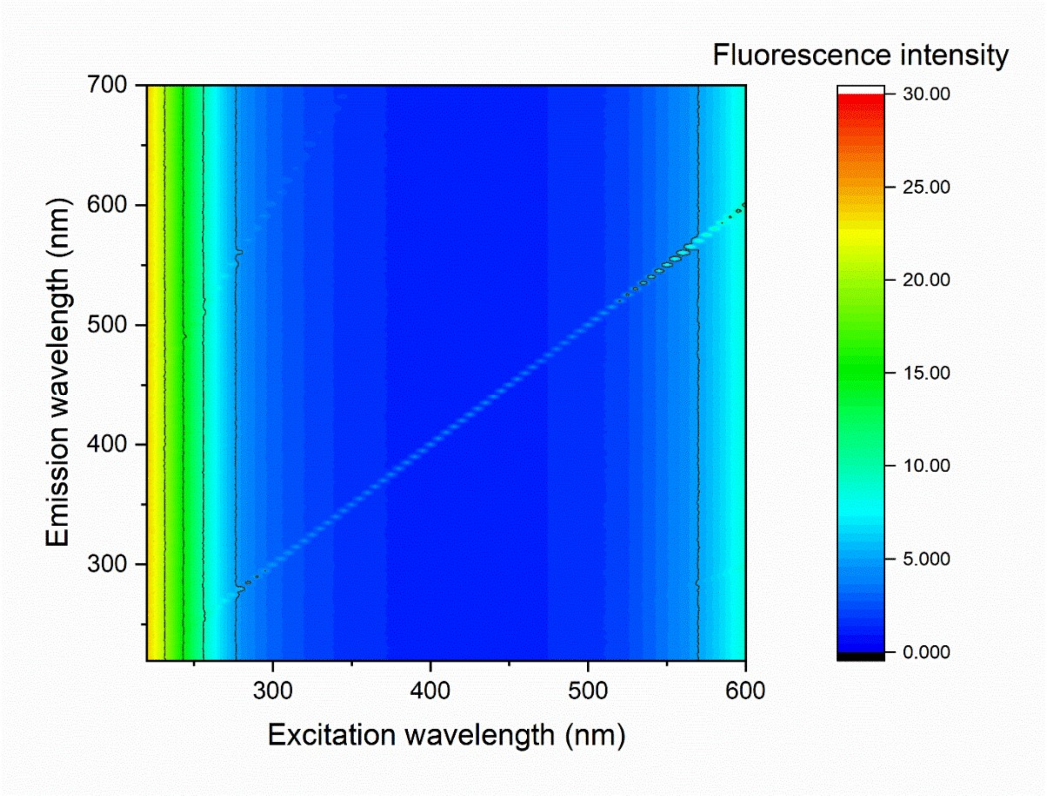

(b)

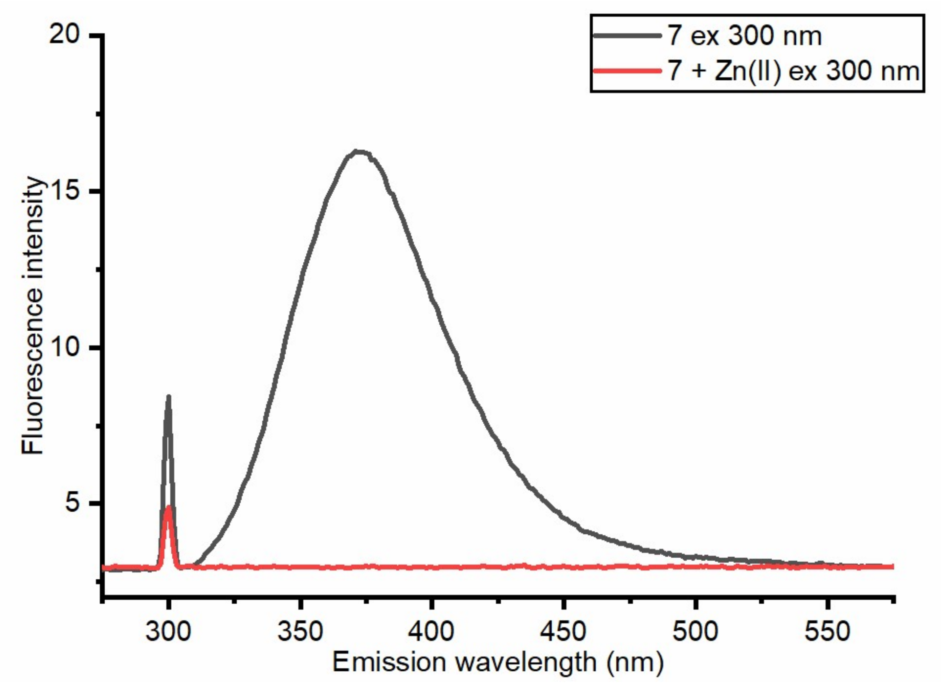

(c)

Since the measurements were made under identical conditions, the fluorescence of **7** can be seen to be quenched by the addition of Zn(II) Cl_2.

3D Fluorescence spectra related to Figure 6 (10 with and without Zn(II)).

(a) 3D Excitation vsEmission spectrum of **10** without Zn(II);.

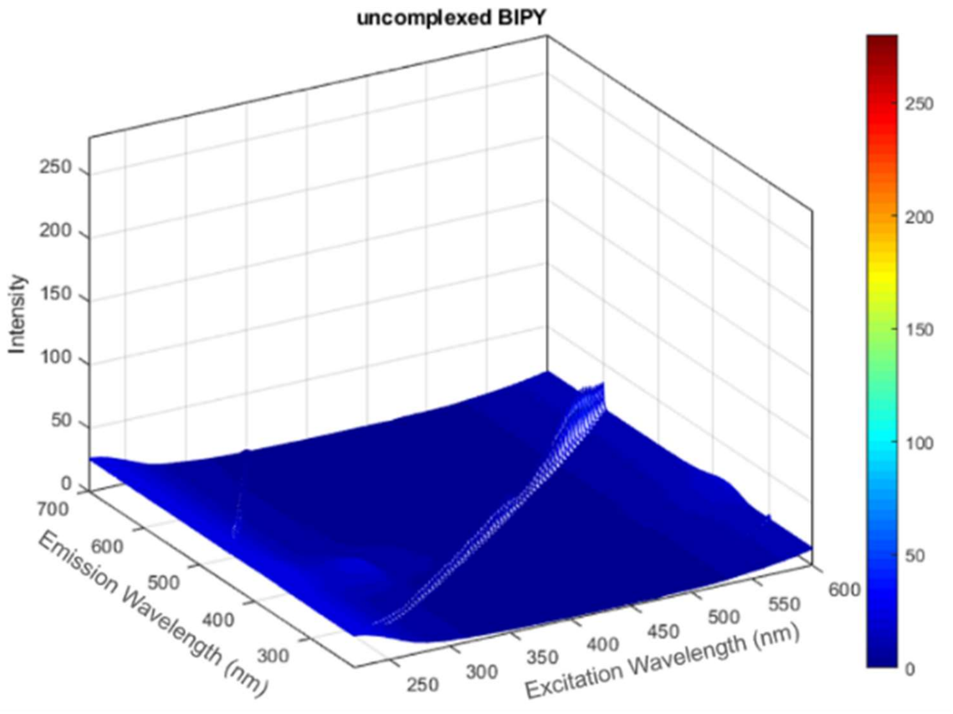

(b) Contour plot of Excitation vs Emission spectrum of **10** without Zn(II).

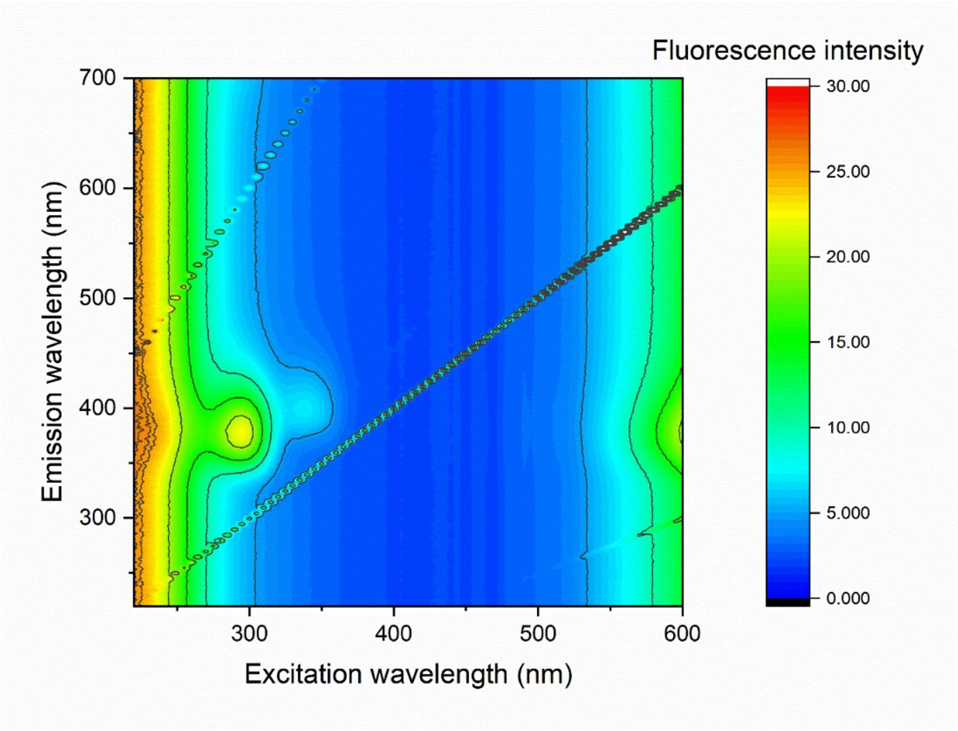

(c) Contour plot of Excitation vs Emission spectrum of **10** with Zn(II) (the 3D spectrum is in the main paper).

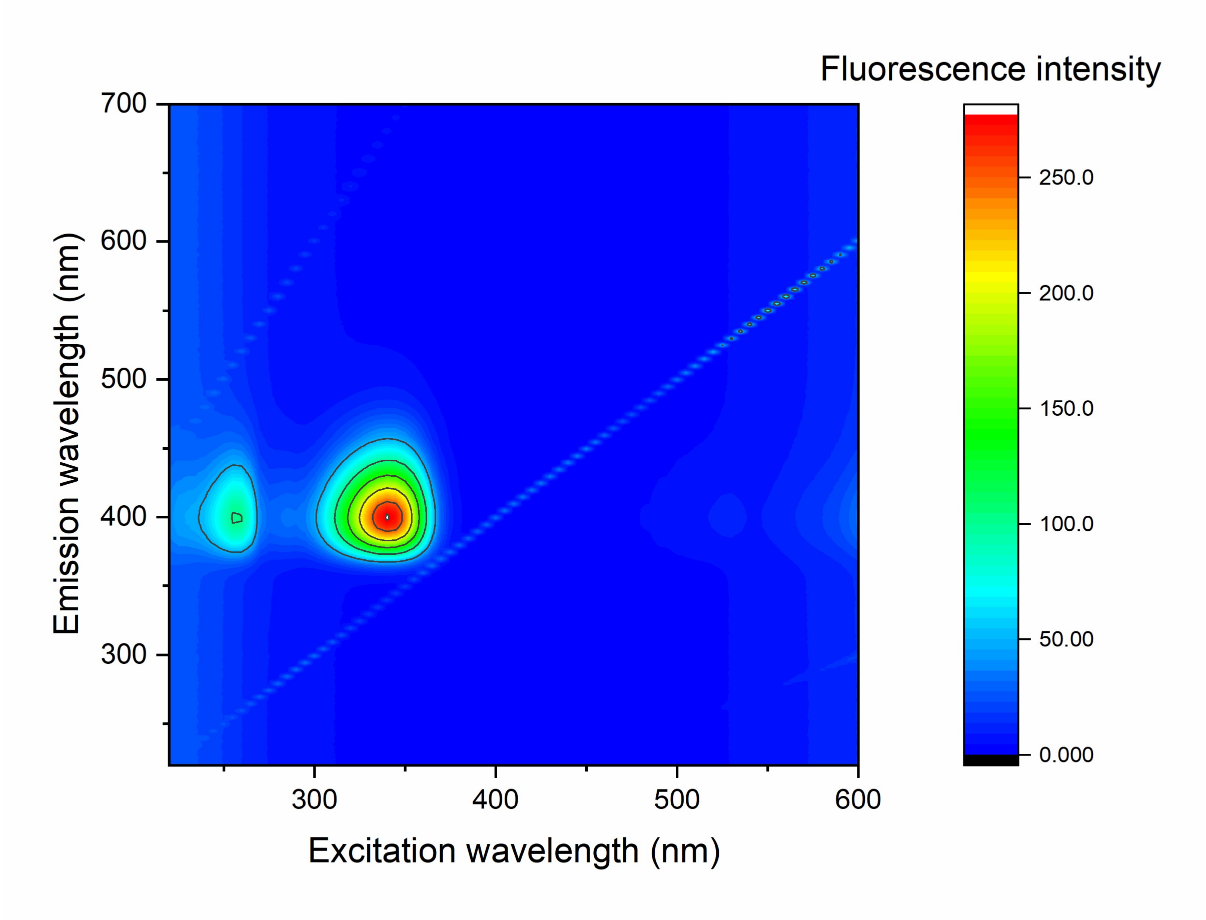

3D fluorescence spectra for other metal complexes (3D and contour):

(a) Co(II)-**10**, (b) Cu(I)- **10**, (c) Cu(II)- **10**, (d) Fe(II)- **10**, (e) Ca(II)- **10**, and (f) Mn(II)- **10** complexes.

(a) Co(II)-**10**:

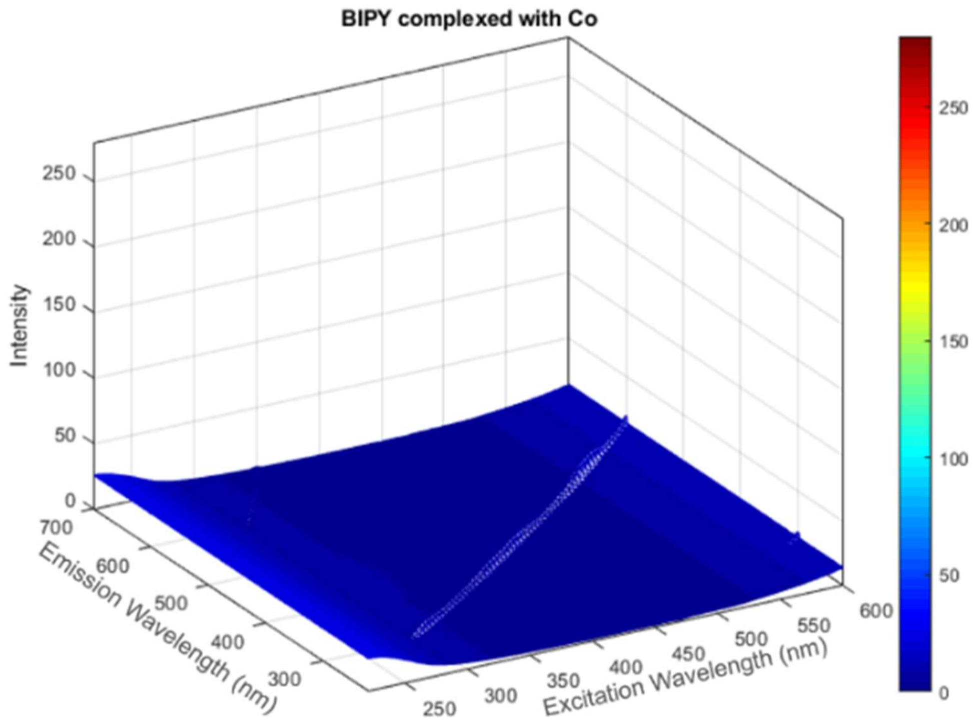

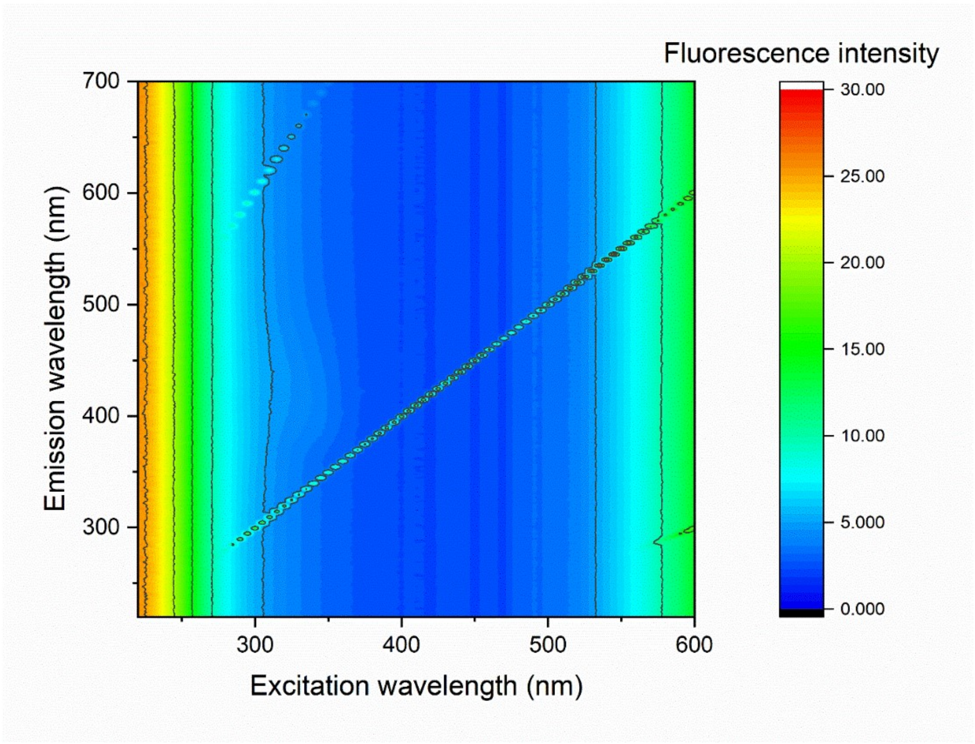

(b) Cu(I)- **10**:

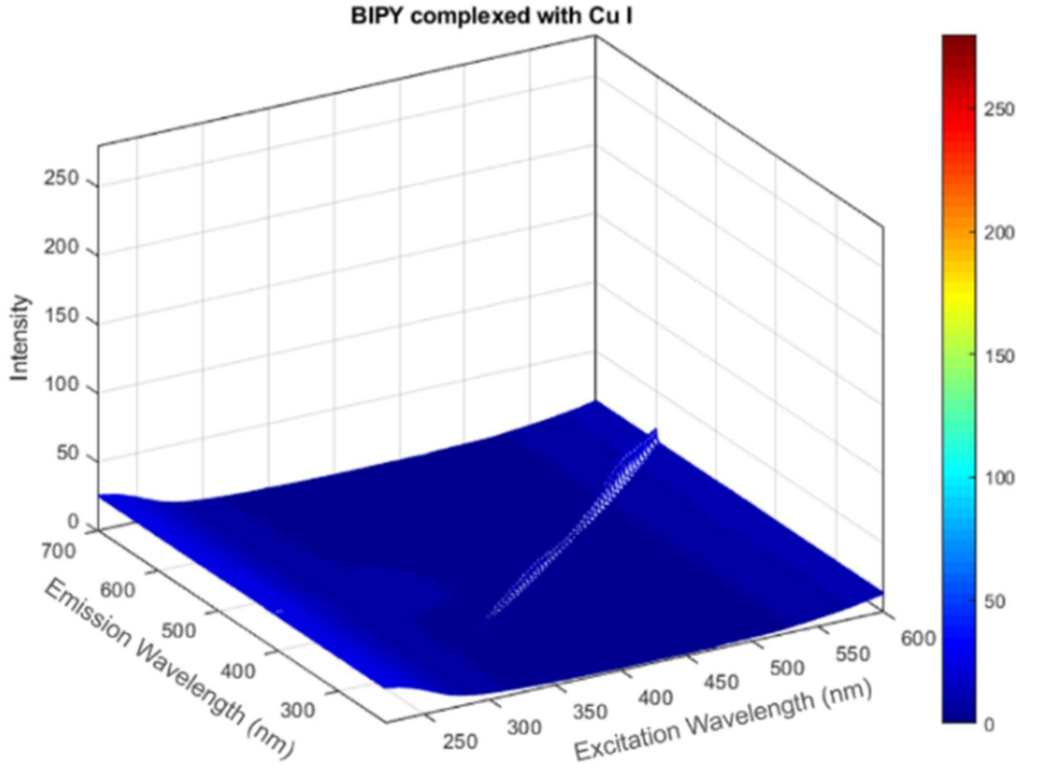

BIPY complexed with Cu I
Intensity
250
200
150
100
50
0
700
600
500
400
300
Emission Wavelength (nm)
250
300
350
400
450
500
550
600
Excitation Wavelength (nm)
250
200
150
100
50
0

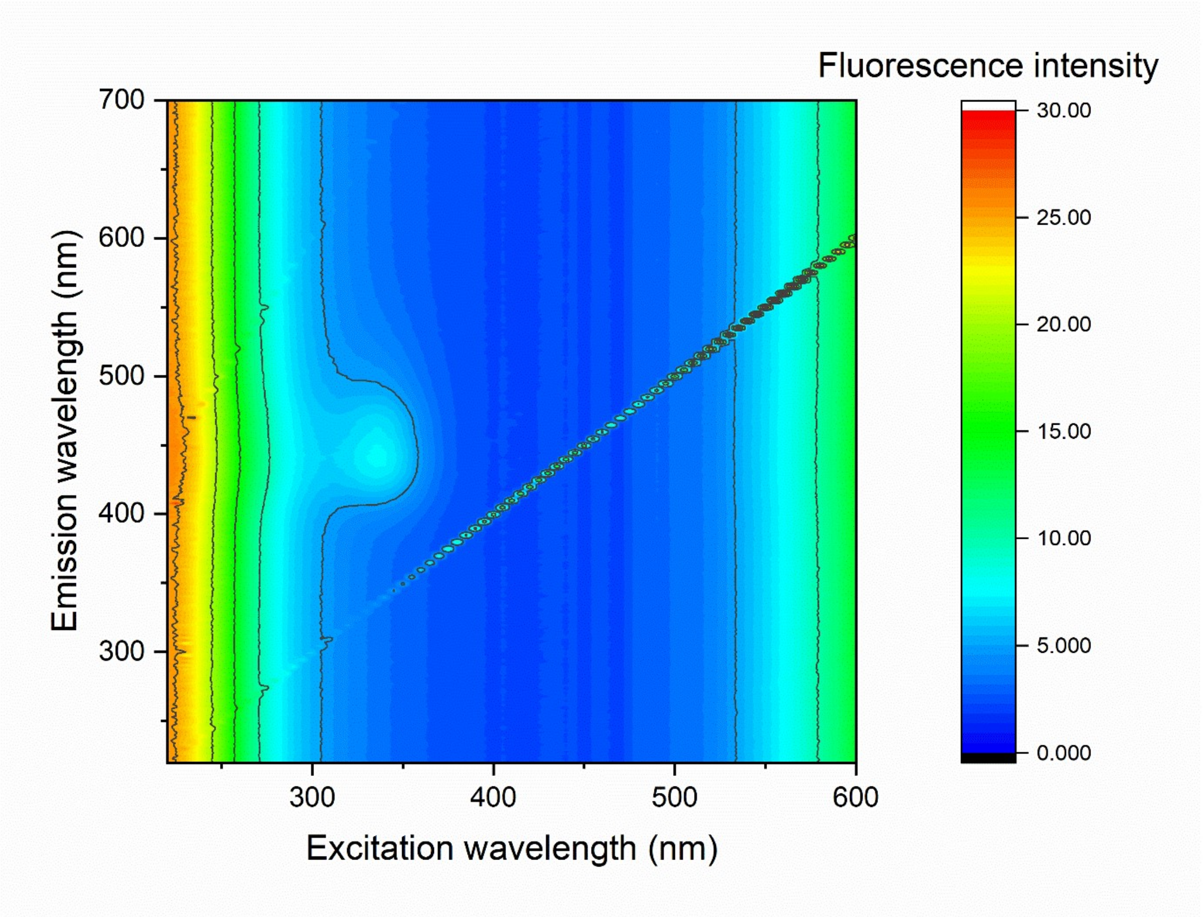

Fluorescence intensity
700
600
500
400
300
Emission wavelength (nm)
300
400
500
600
Excitation wavelength (nm)
30.00
25.00
20.00
15.00
10.00
5.000
0.000

(c) Cu(II)- **10**:

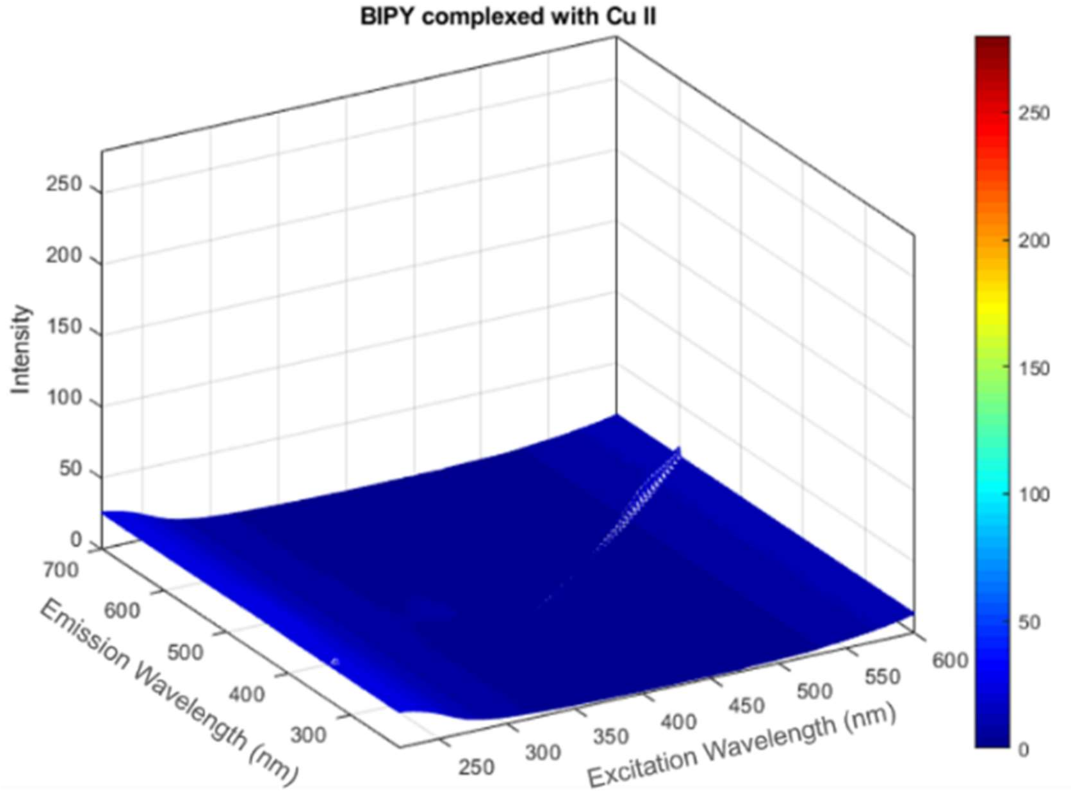
BIPY complexed with Cu II
Intensity
250
200
150
100
50
0
700
600
500
400
300
Emission Wavelength (nm)
250
300
350
400
450
500
550
600
Excitation Wavelength (nm)
250
200
150
100
50
0

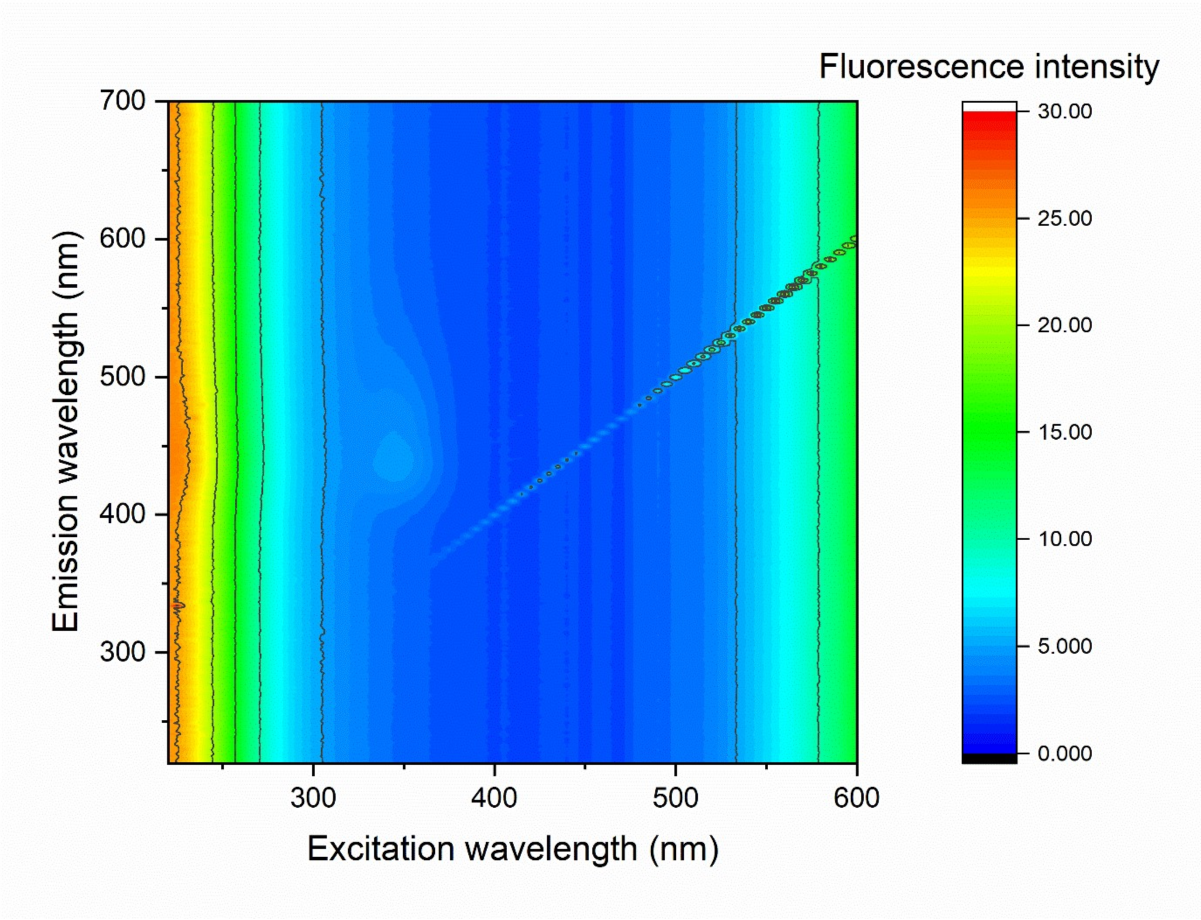
Fluorescence intensity
30.00
25.00
20.00
15.00
10.00
5.000
0.000
700
600
500
400
300
Emission wavelength (nm)
300
400
500
600
Excitation wavelength (nm)

(d) Fe(II)- **10**:

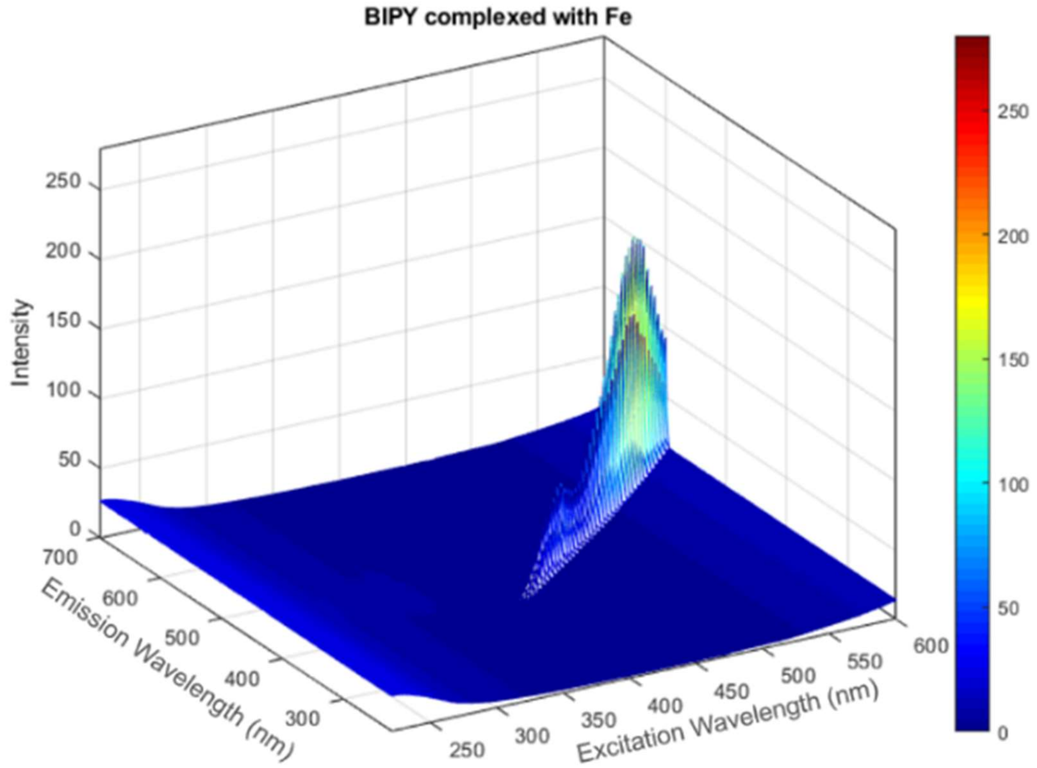

BIPY complexed with Fe
Intensity
250
200
150
100
50
0
700
600
500
400
300
Emission Wavelength (nm)
250
300
350
400
450
500
550
600
Excitation Wavelength (nm)
250
200
150
100
50
0

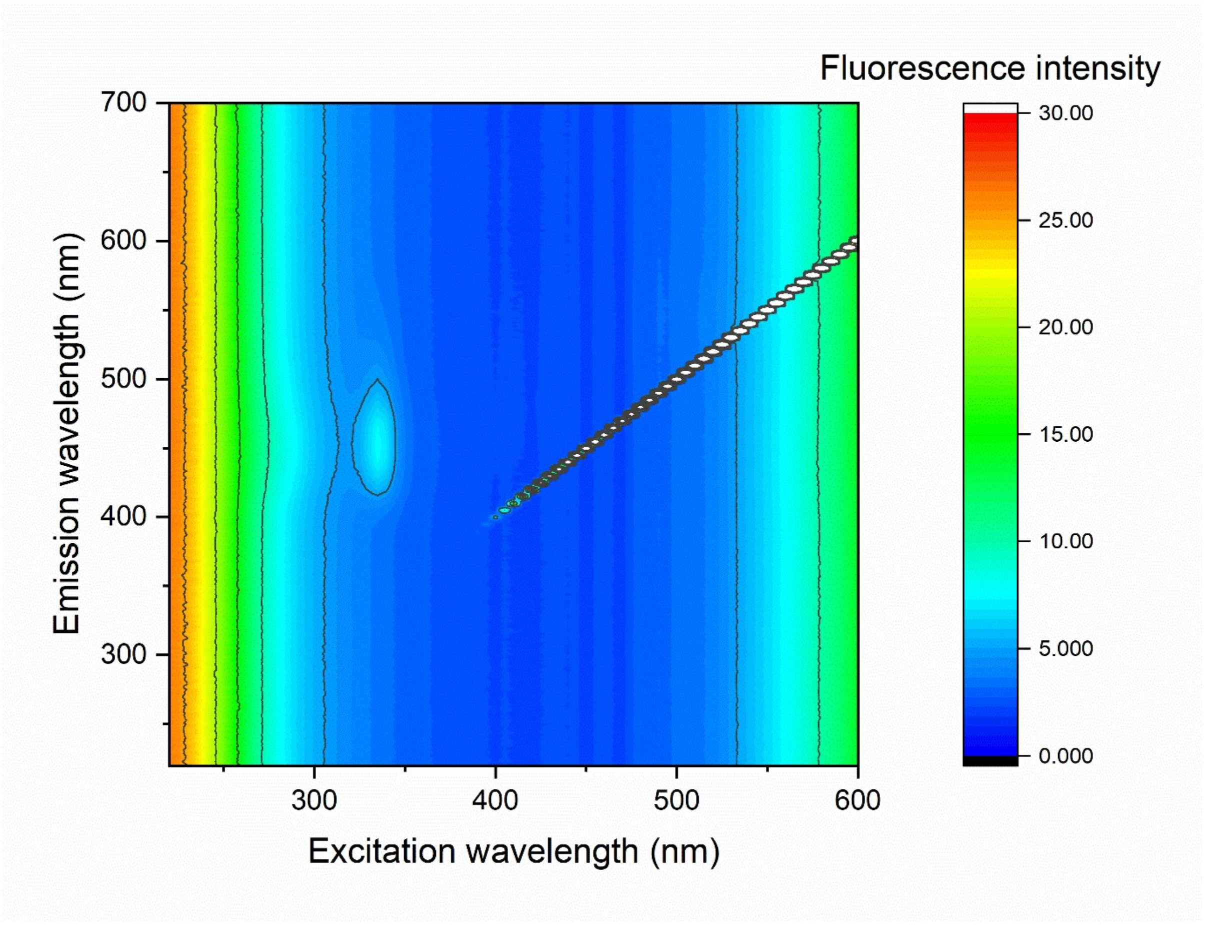

Fluorescence intensity
700
600
500
400
300
Emission wavelength (nm)
300
400
500
600
Excitation wavelength (nm)
30.00
25.00
20.00
15.00
10.00
5.000
0.000

Contour plots of excitation vs emission related to Figure 8 (diol 8)

(a) Compound **8** without Zn(II):

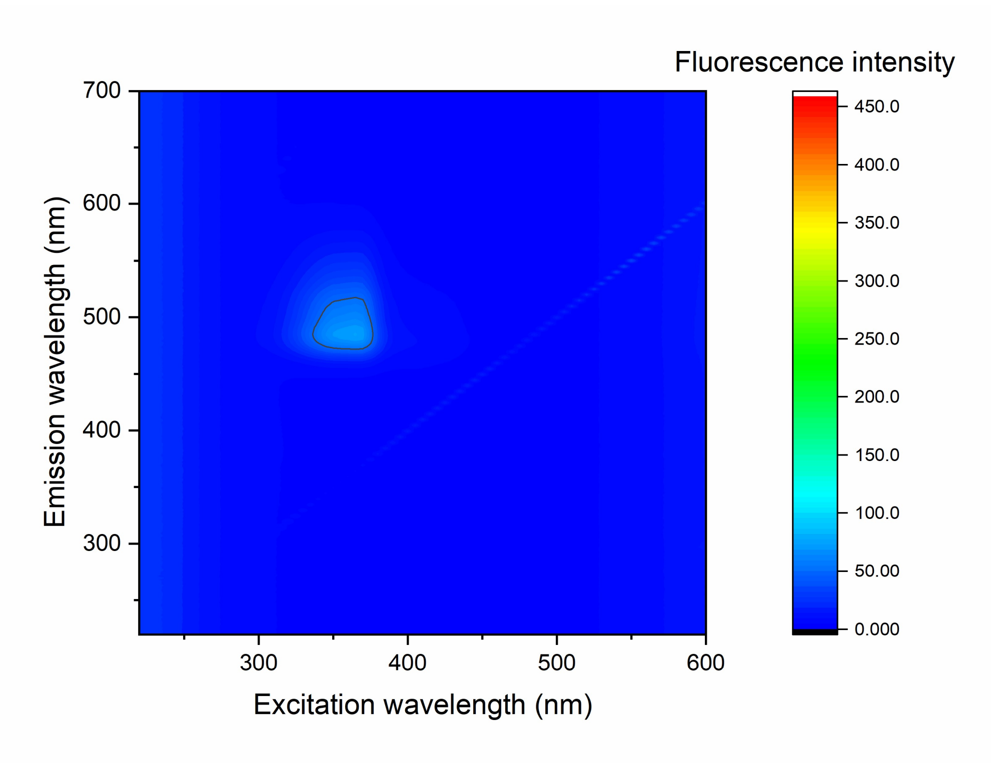

(b) Compound **8** without Zn(II) rescaled:

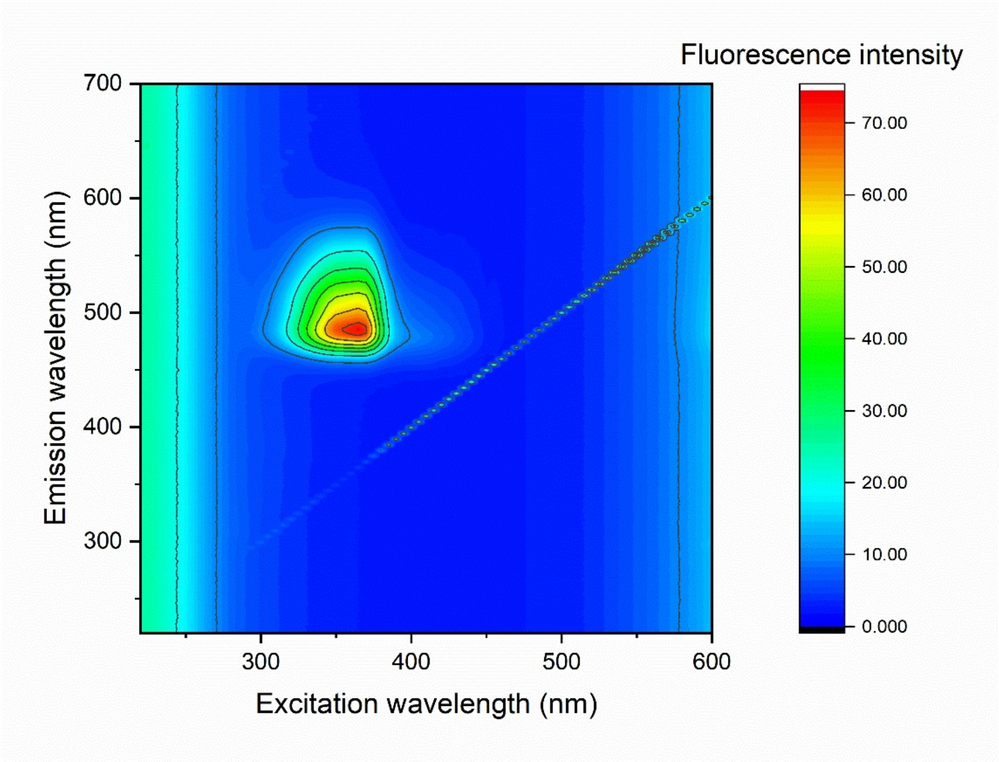

(c) Compound **8** with Zn(II) (the 3D spectrum is in the main paper):

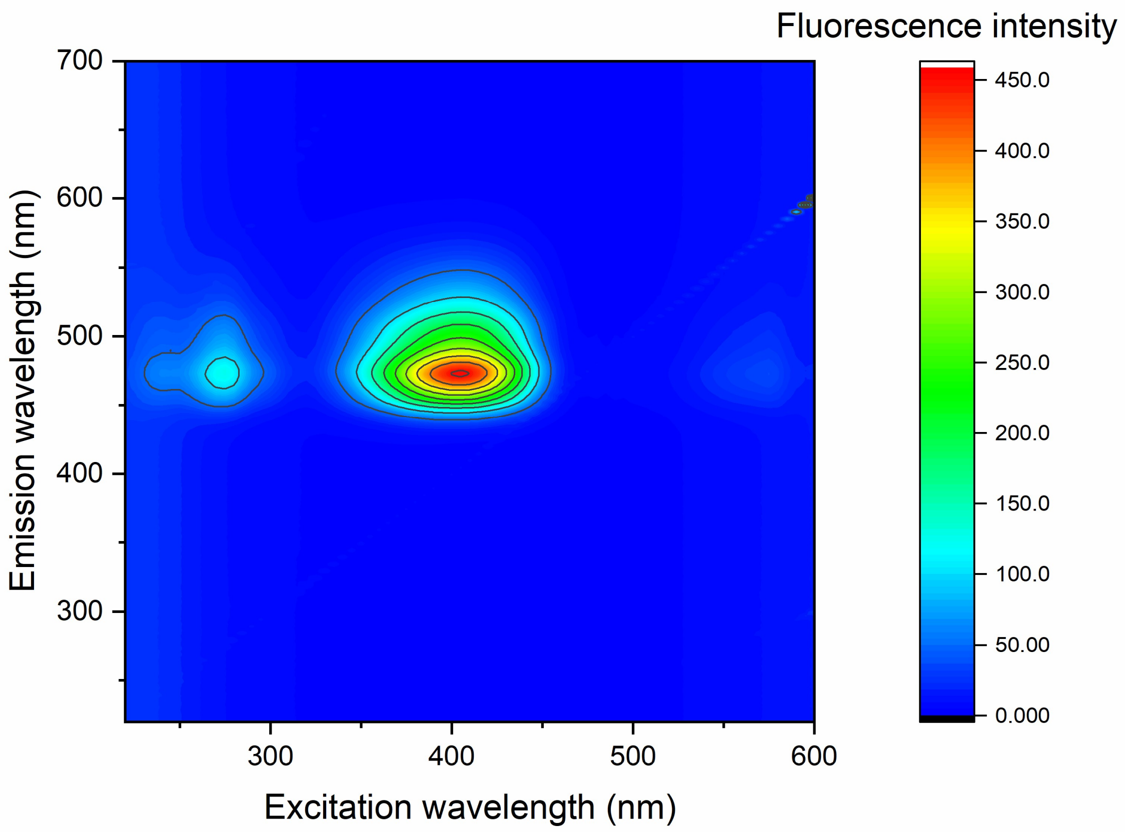

Supplement: RA-009-C9RA06866J-s001 [file RA-009-C9RA06866J-s001.pdf]
